# Human Serum/Plasma Glycoprotein Analysis by ^1^H-NMR, an Emerging Method of Inflammatory Assessment

**DOI:** 10.3390/jcm9020354

**Published:** 2020-01-27

**Authors:** Rocío Fuertes-Martín, Xavier Correig, Joan-Carles Vallvé, Núria Amigó

**Affiliations:** 1 Biosfer Teslab SL, 43201 Reus, Spain; rociofrtsm@gmail.com (R.F.-M.); namigo@biosferteslab.com (N.A.); 2 Metabolomic s platform, IISPV, CIBERDEM, Rovira i Virgili University, 43007 Tarragona, Spain; 3 Lipids and Arteriosclerosis Research Unit, Sant Joan de Reus University Hospital, 43201 Reus, Spain

**Keywords:** glycoprotein, GlycA, NAC, NAG, ^1^H-NMR, inflammation

## Abstract

Several studies suggest that variations in the concentration of plasma glycoproteins can influence cellular changes in a large number of diseases. In recent years, proton nuclear magnetic resonance (^1^H-NMR) has played a major role as an analytical tool for serum and plasma samples. In recent years, there is an increasing interest in the characterization of glycoproteins through ^1^H-NMR in order to search for reliable and robust biomarkers of disease. The objective of this review was to examine the existing studies in the literature related to the study of glycoproteins from an analytical and clinical point of view. There are currently several techniques to characterize circulating glycoproteins in serum or plasma, but in this review, we focus on ^1^H-NMR due to its great robustness and recent interest in its translation to the clinical setting. In fact, there is already a marker in H-NMR representing the acetyl groups of the glycoproteins, GlycA, which has been increasingly studied in clinical studies. A broad search of the literature was performed showing a general consensus that GlycA is a robust marker of systemic inflammation. The results also suggested that GlycA better captures systemic inflammation even more than C-reactive protein (CRP), a widely used classical inflammatory marker. The applications reviewed here demonstrated that GlycA was potentially a key biomarker in a wide range of diseases such as cancer, metabolic diseases, cardiovascular risk, and chronic inflammatory diseases among others. The profiling of glycoproteins through ^1^H-NMR launches an encouraging new paradigm for its future incorporation in clinical diagnosis.

## 1. Background

Plasma glycoproteins belong to the large family of acute-phase proteins (APPs), which are directly related to inflammatory disorders [1]. Research in this area is expanding as several studies suggest that the change in the concentration of APPs and the pattern of glycation of these proteins can influence cellular changes in a large number of diseases [2,3,4] so they can be regarded as diagnostic markers [5].

It should be noted that glycoproteome analysis is much more complex than proteome analysis because, unlike proteins in which amino acid sequences are unique, oligosaccharides and polysaccharides from glycans are normally composed of an enormous diversity of both linear and branched sugar residues, which increase the complexity of the glycoprotein structures [6]. For this reason, the analysis of glycoproteins is often a technical challenge. In recent years, nuclear magnetic resonance (NMR) has played a major role as an analytical tool for metabolomic studies with biological fluids, especially for serum and plasma samples. A clear example of this is its application for the determination of lipoproteins [7]. Unlike other techniques, NMR is capable of quantifying metabolites in a reproducible and effective way, so it is widely used in large epidemiological studies and has started to be introduced in routine clinical practice [8]. In this review, we will focus on NMR as a promising technique for quantifying glycoproteins in serum or plasma.

This review reflects the growing interest in determining proton NMR (^1^H-NMR) circulating glycoproteins and their application in the clinical field, which may pave the way for these markers to be used in the prediction and monitoring of several diseases.

## 2. Glycoproteins: A Biochemical Approach

Proteins can undergo numerous chemical modifications in their structure which have important modulating effects on their biological function, alter their cellular location and capacity to interact with other proteins, or even determine their own degradation. These modifications occur in proteins once they have synthesized and are called post-translational modifications (PTMs).

PTMs increase the functional diversity of the proteome. These modifications include phosphorylation, myristoylation, farnesylation, cysteine oxidation, ubiquitination, acetylation, phosphorylation, glycosylation, methylation, nitrosylation, etc., and influence almost all aspects of normal cell biology and pathogenesis [9]. Glycosylation is the addition of one or more chains of carbohydrates (glycans) to a protein. This is the main chemical modification of most plasma-membrane and secretory proteins [10]. Glycoproteins participate in many key biological processes including cell adhesion, molecular trafficking and clearance, receptor activation, signal transduction, and endocytosis. Most of the proteins in blood plasma (except for albumin) are highly glycosylated, and the glycosylation of these and other secreted proteins can provide solubility, hydrophilicity, and negative loading, thus reducing unwanted intermolecular interactions and protecting them against proteolysis or simply varying their function. Cell surface membrane proteins, such as receptors, adhesion molecules, and channels, are also typically glycosylated, and this modification can also change their function [11].

**Figure 1:**
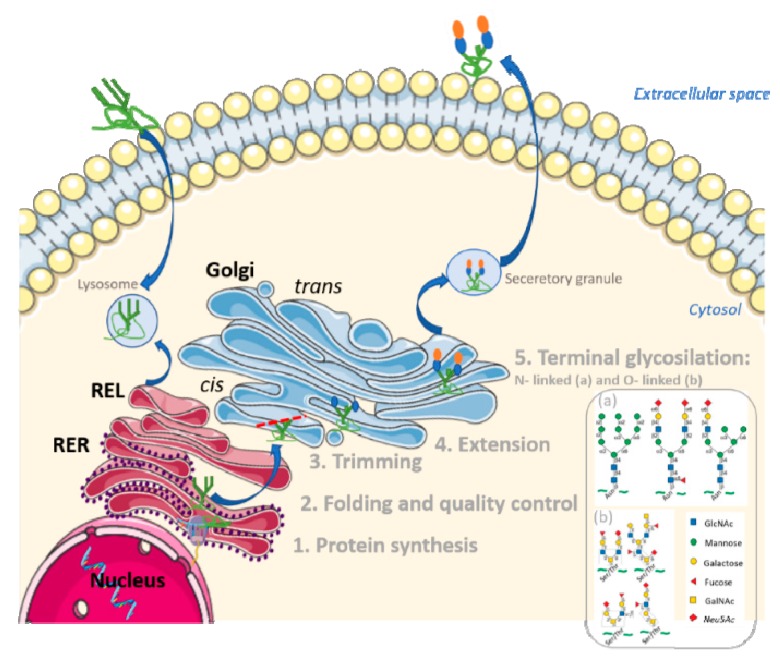
Initiation and maturation of glycoproteins in the ER–Golgi–plasma (Endoplasmic reticulum–Golgi–plasma) membrane pathway. This illustration outlines an overview of the mechanisms for initiation, trimming, and elongation of the glycoprotein in a human cell. Orange and blue spheres represent the addition of glycans chains to proteins (in green) in the Golgi apparatus. Examples of *N*-glycans structures (**a**) and *O*-Glycans structures (**b**) are also represented.

Glycosylation is regarded as the most complex PTMs because of the large number of enzymatic steps involved [12]. Most of the proteins secreted in eukaryotic cells are translocated to the endoplasmic reticulum (ER) where they are folded, modified, and subjected to quality control mechanisms. The protein component of all glycoproteins is synthesized in the rough ER (RER). They then make their way through multiple stacks of the Golgi apparatus (from *cis* Golgi to *trans* Golgi), finally being distributed to various destinations from the *trans* Golgi network. This is where the addition of glycans to the polypeptide chain of the protein occurs by complex dynamic interactions between hundreds of enzymes such as glycosyltransferase reactions, enzymes that transfer activated forms of monosaccharides from nucleotide sugars, and lipid-linked sugar intermediates to acceptors including proteins, lipids, and growing glycan chains [11]. Some of these activated forms of monosaccharides are mannose, fucose, galactose, *N*-Acetylglucosamine (GlcNAc), *N*-Acetylgalactosamine (GalNAc), *N*-acetylneuraminic acid (Neu5Ac) or sialic acid, among others. Figure 1 shows an overview of the cellular organization of glycosylation.

Protein glycosylation encompasses *N*-glycans, *O*-glycans, and glycosaminoglycans (frequently referred to as proteoglycans) [13]. *N*- and *O*-glycosylation are the most commonly detected types. The structures of *N*- and *O*-linked oligosaccharides are very different, and different sugar residues are usually found in each type [14]. In all *N*-linked oligosaccharides, GlcNAc is linked to the amide nitrogen of the asparagine (Asn) of a consensus peptide sequence Asn-X-Ser [15] (with X being any amino acid except proline) and they always contain mannose. This glycosylation usually has several branches, each terminating with a negatively charged Neu5AC or sialic acid residue [13,14,16]. However, *O*-linked oligosaccharides are linked to the hydroxyl group of serine (Ser) or threonine (Thr) via GalNAc or (in collagens) to the hydroxyl group of hydroxylysine via galactose and they are generally short, often containing only one to four sugar residues [13,14,16].

It is important to note that *O*-glycosylation is more abundant intracellularly and has been associated mostly with protein signaling and intracellular mechanisms, while *N*-glycosylation is predominant in circulating proteins [17]. In addition, *N*-glycan synthesis can be easily altered by pathophysiological conditions such as inflammatory and autoimmune diseases and in the pathophysiological process of aging, which is why *N*-glycans are emerging as powerful and reliable biomarkers of several diseases [15] as we shall see below.

## 3. Clinical Importance of Glycoproteins

Both *N*-glycans and *O*-glycans play an important role in the functions of the glycoprotein involved in various cell recognition signals and pathological situations [13,18,19,20,21,22,23], so they are potentially potent and reliable biomarkers of various diseases. Because of the large number of biological processes in which glycans participate, it is not surprising that defects in the synthesis of glycans can be the direct cause of numerous diseases and, therefore, markers of the disease [5]. It was not until the 1980s that NMR began to arouse great interest in the search for clinically relevant markers, including APPs [24]. Since before 1987, it has been reported that the concentration of plasma glycoproteins changes in a number of clinical disorders characterized by inflammation (e.g., different types of cancer, rheumatoid arthritis, some liver diseases, trauma, etc.) and pregnancy [25,26,27,28,29]. The altered synthesis of *N*-glycans is thought to underlie these pathological conditions [15]. It is important to note that most serum glycoproteins have *N*-linked, and less frequently *O*-linked, sugars in their structure. Nonetheless, there is still much to learn about the role of glycans in disease mechanisms. However, as more information on protein glycation emerges, it becomes increasingly clear that glycation is strictly regulated and that the binding of glycan to proteins is of paramount physiological importance [30].

In general, the most studied glycoproteins of clinical importance are the glycoproteins of the cell membrane, whose glycans, called glycocalyx, play important roles in the immune response. For example, selectins are a widely studied family of membrane proteins that are glycosylated and play a crucial role in the recruitment of leukocytes, the onset of the immune response and the onset of inflammation [31]. It has been shown that the deregulation of selectins or their glycoprotein ligand are associated with atherosclerosis, thrombosis, and even the metastasis of tumors [32].

We want to place special emphasis on the glycoproteins of human plasma, which in recent years have been attracting considerable interest as possible biomarkers of disease [33]. Human plasma glycoproteins belong to the large family of APPs, which are characterized by increasing or decreasing their concentration (positive or negative acute-phase proteins respectively) by up to 25 percent during inflammatory disorders [1]. Most of these APPs are glycosylated proteins secreted from hepatocytes [34]. The alterations in the glycosylation of these proteins indicate cellular changes in a large number of diseases, which is why they can be regarded as diagnostic markers of a disease. Numerous changes in the glycosylation of serum proteins have been reported for inflammatory diseases. Table 1 shows some examples of glycation changes in serum glycoproteins that have been associated with various inflammatory diseases.

## 4. Measurement Techniques for Glycoprotein Determination

### 4.1. Traditionally Used Techniques to Measure Glycated Proteins

Various techniques have been used to measure individual glycated proteins. Examples of these are lectin analysis [68], DNA sequencing equipment-fluorophore assisted carbohydrate electrophoresis (DSA-FACE) [15], chromatographic methods such as high performance liquid chromatography (HPLC) [69], or hydrophilic interaction liquid chromatography (HILIC) [68], MALDI-TOF analysis of tryptic glycopeptides [68], electrophoresis [26], mass spectrometry (MS) [70], or the recently developed glycoblotting method that combines the BlotGlycoABC bead and MALDI-TOF MS to detect abnormal glycosylation patterns in the whole serum glycoproteins [71,72]. In a recent review, A. Conelly et al. described the current assays of glycoproteins in biological fluids, among which were the enzyme-linked immunosorbent assays (ELISAs), electrochemiluminescence immunoassay (ECLIA), Luminex-based assays, radioimmunoassays (RIA), and nephelometric assays, which quantify the amount of protein in biological samples [73].

Most of these techniques measure a specific individual glycoprotein. However, measuring the glycan portion of inflammatory proteins is becoming increasingly useful for diagnostic purposes [73]. Some of the newest high-performance techniques such as mass spectrometry (MS) and nuclear magnetic resonance spectroscopy (NMR), which have recently been introduced to the clinical laboratory, have been used for this purpose.

It is important for techniques to be cost-effective so throughput must be high and molecular measurements absolute. For this reason, MS and NMR have been the most used techniques. MS is used for more detailed characterizations and is based on mass difference, but it produces complicated spectroscopic data and is expensive. NMR, however, is currently the only methodology capable of providing reproducible quantifications of high-performance metabolites in a cost-effective manner [8]. It has also been widely used in recent years to quantify lipoproteins in a fully optimized way and has generated considerable medical advances [7]. Although NMR is not as specific as MS, one major advantage is that in a very short time it can provide a complete metabolic profile of a serum or plasma sample [8].

In this review, we focus on proton nuclear magnetic resonance (^1^H-NMR) applications based on methods for quantifying circulating glycoproteins. The great versatility of NMR and the integrated computational methods of systems biology provides robust and reliable tools for biomedical research.

### 4.2. Serum/Plasma NMR Glycoprotein Analysis

As mentioned in the section above, measuring lipoproteins in plasma and serum has been a key issue in recent years. Similarly, the measurement of glycoproteins is becoming increasingly important, particularly because they can be quantified from the same experiment used for lipoproteins, which means considerable savings and maximum profitability. Another major advantage associated with NMR is the low experimental variability between laboratories. In fact, greater variability is due to interpersonal variability itself and measurements on different days [74].

#### 4.2.1. Sample Handling and Preparation

Serum and plasma fractions are taken from blood samples that have undergone various biochemical protocols after collection [75]. In the case of serum, coagulation factors (i.e., fibrinogen) and blood cells are removed by centrifugation, while plasma is typically obtained from blood samples with an added anticoagulant agent (i.e., heparin or EDTA). These agents produce high intensity peaks (EDTA) or overlapping signals (heparin) in the NMR spectra [76], which is why for some NMR applications, serum may be preferred to plasma. However, the metabolic profiles detected in plasma and serum by NMR are comparable, although signals from EDTA complicate the plasma spectrum profile [77].

One major advantage of NMR in the study of plasma and/or serum is that measurements can often be made with minimal sample preparation. Normally, in NMR experiments on biofluids samples only require the addition of phosphate buffer in a small volume of deuterated solvent, and an internal standard for chemical shift reference and quantitative normalization.

#### 4.2.2. Sample Storage

For the analysis of lipoproteins and other plasma/serum metabolites by NMR, some storage issues have to be considered. Samples can be stored in good refrigerated conditions for several days at 2–4 °C, and up to 7 days at temperatures below 4 °C [78]. They can be successfully stored at −20 °C for a moderate period of time (up to 1–2 months), but some enzymes, such as plasma esterase, may still be active at this temperature. Therefore, for longer storage periods, −70 or −80 °C is required [79]. In terms of stability, some studies report a high degree of stability of glycoproteins measured by NMR in frozen samples and stored for more than 10 years [80].

However, the protocols established for the preservation of serum and plasma samples may be used in order to minimize a possible variability in the results obtained by different analytical platforms [76,81].

#### 4.2.3. Processing of NMR Spectra for Glycoprotein Profiling

**Figure 2:**
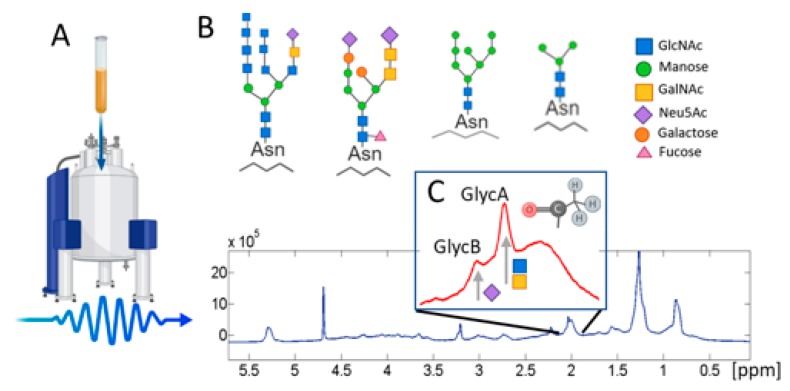
^1^H-NMR (nuclear magnetic resonance) glycoprotein analysis methodology. (**A**) Sample tube and spectrometer; (**B**) examples of *N*-glycans with different residues attached to the protein chain by asparagine (Asn); (**C**) ^1^H-NMR spectrum produced by the sample in which the region of the glycoproteins is marked. The chemical group producing this signal is indicated.

The procedure for obtaining the spectrum before quantification of the glycoproteins has been extensively described [7]. Briefly, the spectrum goes through several phases before it goes for glycoprotein quantification. First, it is obtained by applying one or more pulse sequences. Generally, the most standard pulses are the nuclear Overhauser effect spectroscopy (NOESY)-presaturation sequence that acquires a quantitative serum spectrum by suppressing the water peak, the Carr–Purcell–Meiboom–Gill (CPMG) pulse sequence that acquires low molecular weight metabolites, a diffusion-edited pulse sequence with bipolar gradients, and finally, the longitudinal eddy-current delay (LED) with presaturation of the water signal. Most authors use a single pulse for glycoprotein profiling, the most common being CPMG, although LED or NOESY are also used, as can be seen in Table S1 of the Supplementary Material. The next step is to apply an algorithm to quantify the region in which glycoproteins resonate. Several algorithms have been described in the literature; binning, peak alignment, and combinations of peak alignment and data reduction such as PARS, the curve-fitting algorithm, the peak alignment tools in HiRes, and targeted profiling [82]. Figure 2 shows a scheme of the analysis of glycoproteins by ^1^H-NMR.

In the case of glycoproteins, the region is a composite signal with a prominent peak centered at approximately 2.03 ppm of the ^1^H-NMR spectrum. This signal is produced by the -COCH_3_ acetyl groups of *N*-acetylglucosamine and *N*-acetylgalactosamine and *N*-acetylneuraminic acid [83]. The resonances of the sugar ring protons of the glycoproteins are not clearly discernible in the plasma spectra because of extensive overlap with the more intense signals from glucose [24].

Otvos et al. were the first to call the main peak of this signal “GlycA” (glycoprotein acetylation) in 2015 [84]. Before this, other authors had referred to this same peak as *N*-acetyl glycoprotein (NAC or NAGs) or *N*-Acetyl glucosamine (NAG). The low detection sensitivity of NMR means that species present at concentrations less than about 20 µmol/L are undetectable under the conditions of measurement. So, only a small subset of acute phase glycoproteins make meaningful contributions to the GlycA signal [84]. It appears that measured GlycA concentrations are mainly due to contributions from α1-acid glycoprotein, haptoglobin, α1-antitrypsin, α1-antichymotrypsin, and transferrin [24,84]. With the exception of transferrin, the circulating concentrations of the proteins that constitute the GlycA signal increase during the acute-phase response [80]. This heterogeneous composition is a challenge for future research since NMR alone cannot accurately measure the concentration of each of the individual proteins in the signal. However, complementary studies using NMR and other techniques such as immunoassays and even machine learning techniques have shown that α1-acid glycoprotein is the major contributor to the signal, followed by α1-antitrypsin [24,80,84,85].

During the present decade, several research groups have focused on developing methods to determine ^1^H-NMR glycoproteins. Some companies that specialize in the analysis of other metabolites, such as lipoproteins, have expanded their services by also offering the analysis of glycoproteins since, as mentioned above, they are obtained from the same spectrum. A clear example of this is the NMR-algorithm (Lipoprofile^®^) at LabCorp, Inc. (formerly LipoScience, Inc.), which quantifies GlycA in plasma [84]. Another example is Nightingale Health Ltd. (formerly Brainshake, Ltd.), which also quantifies GlycA in serum samples [86]. Also noteworthy is Biosfer Teslab S,L (Liposcale^®^). This company recently developed a method for analyzing glycoproteins by ^1^H-NMR spectra analysis which obtains parameters other than GlycA such as GlycB (concentration of acetyl groups of *N*-acetylneuraminic acid) and ratios H/W GlycA and H/W GlycB, which provide information on the function in each case. This function depends on its height, which is related to the concentration, and its width, which is related to the flexibility and the aggregation of the molecules generating the signal. Higher and narrower signal peaks have been related to some inflammatory pathologies [83]. The glycoprotein profiling methods mentioned are described as glycoprotein profiling methods in the Supplementary Material.

## 5. ^1^H-NMR Glycoprotein Clinical Studies

Although we have seen that several changes in the glycation of proteins give rise to disease and that they can be measured by several techniques, in this review we focus on the most recent conclusions in the literature on glycoprotein studies, particularly those drawn from ^1^H-NMR studies.

**Figure 3:**
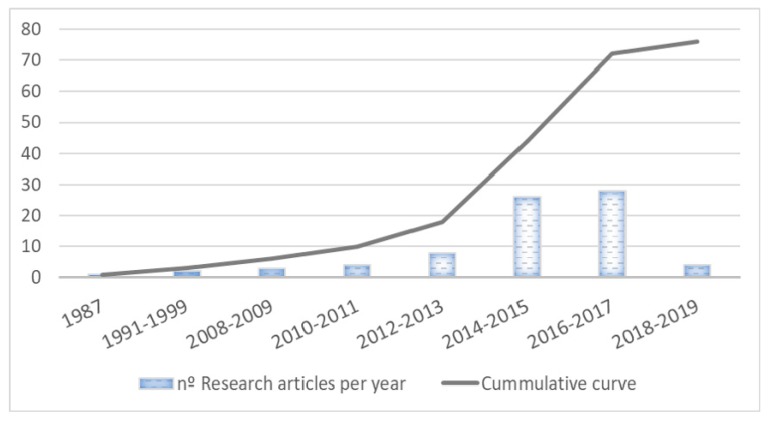
Trend graph of the number of research articles per year in recent year.

A broad search of the literature was performed using Elsevier’s scientific database SCOPUS, PubMed, and the Google scholar database. SCOPUS (with cut-off dates between 1999 and 2019) was the main database searched and the following search terms were used: “Glycoproteins OR GlycA OR Glyc A” AND “NMR OR nuclear magnetic resonance OR H-NMR” AND “serum OR plasma” AND “marker OR biomarker”. The search was completed with manual reference checks on Google Scholar and the PubMed database with the same keywords. The initial result of the search was 239 documents that were subsequently filtered by species (humans), English language, and fields of no interest such as agricultural sciences. Finally, we discarded the articles that spoke of specific glycoproteins not detected by ^1^H-NMR and articles that included NMR but only referred to lipids. As shown in the trend graph (Figure 3), the detection of glycoproteins by ^1^H-NMR has been a topic of great interest in recent years and the number of studies is progressively increasing.

### 5.1. Former Studies on ^1^H-NMR Detection and Identification of Glycoproteins

Prior to 1990, the literature search offered little clinical data on the detection of glycoproteins or glycans by ^1^H-NMR. However, some early works in 1983 and 1984 by Nicholson and colleagues reported assignments to peaks in the ^1^H-NMR spectrum [87,88] and mentioned the assignment of the spectrum peaks for *N*-acetyls from sugars of glycoproteins [87]. The first studies on the characterization of glycoproteins were carried out by Bell et al. in 1987 and determined the *N*-acetyl protons of highly mobile *N*-acetylated carbohydrate side-chains associated with plasma glycoproteins (mainly α1-acid glycoprotein, α1-antitrypsin, haptoglobin, transferrin, and immunoglobulins) [24]. In 1999, when even the signals of the *N*-acetyl groups in the ^1^H-NMR spectrum were not unequivocal, one of the first studies compared the glycoprotein signals with the levels of immunoglobulins detected by other biochemical methods [77]. It was then theorized that both analytical approaches should be used with different strategies because NMR parameters were more suitable for longitudinal studies of chronic situations [77]. These previous studies suggested that glycoproteins of acute-phase reactants, which reflect both acute and chronic inflammation, may be useful for the detection, prognosis, and therapeutic monitoring of tissue damage marked by inflammation in several pathologies.

### 5.2. Clinical Applications 

^1^H-NMR has been used to characterize glycoproteins in several pathologies since the first studies mentioned above. This section describes the findings on changes in protein glycation on the different clinical study topics that have been described in literature divided in (a) diseases; (b) treatment effects and lifestyle; and (c) other conditions. A summary of the main findings on each topic can be found in Table 2,Table 3 and Table 4. A more detailed explanation of each of the studies in this section is reflected in Table S1 of the Supplementary Material.

#### 5.2.1. Diseases

##### Tumors and Cancer

Cancer is the second leading cause of death globally and is estimated to have accounted for 9.6 million deaths in 2018. Biomarker research in this field has been becoming increasingly important in recent years for the diagnosis and prognosis of different kinds of tumors. Evidence in the literature shows that chronic inflammation is a potential factor associated with tumor development [92]. Alterations in glycosylation patterns regulate the development and progression of cancer, potentially serve as important biomarkers and provide a set of specific targets for therapeutic interventions [20]. Changes in glycosylation commonly associated with cancer include sialylation, fucosylation, increased GlcNAc-branching of *N*-glycans, over-expression of truncated mucin type *O*-glycans, and increased circulating *N*-acetyl glycoprotein levels [24]. These changes increase structural glycan heterogeneity and alter the function of cells [145].

It was not until 1988 and 1989 that ^1^H-NMR began to be used as a new tool for studying the structural and metabolic modifications in cancer patients [146]. In recent years, NMR-based body fluid metabolomic studies have been increasingly performed for the diagnosis and prognosis of the disease [147].

Breast cancer (BC) is the most frequent cancer among women. It affects 2.1 million women every year and causes the highest number of cancer-related deaths (WHO, 2018). In BC research so far, metabolomics has been generally used for the direct characterization of tumor metabolism alterations [91]. ^1^H-NMR has been used to demonstrate that serum metabolite profiles derived from metastatic breast cancer (MBC) patients are different from localized early breast cancer patients (EBC). Compared to EBC patients, MBC patients display higher serum concentrations of *N*-acetyl glycoproteins (NAC1 *p* < 0.027 and NAC2 *p* < 0.007) [91]. Suman et al. reinforced these results when they found high levels of NAG among other metabolites such as hydroxybutyrate, lysine, glutamate, glucose, and lactate metabolites in BC patients, which were potentially useful for diagnosing BC progression [93]. However, contrary to these results, in an NMR-based untargeted metabolomic study, Lecuyer et al. reported lower plasma levels of glycoproteins, lipoproteins, lipids, acetone, glycerol-derived compounds, unsaturated lipids, and a higher risk of developing breast cancer within the following decade [148].

Two ^1^H-NMR studies have been carried out in the cystic fluid of ovarian cancer [89,149] but only one of them focuses on the *N*-acetyl groups of glycoproteins and shows that they are positively associated with the pathology [89]. Studies on cervical squamous cell carcinoma (CSCC) have also shown an increase in the glycoprotein peak of the ^1^H-NMR plasma spectrum, as well as the peak of other metabolites, compared with patients with cervical intraepithelial neoplasia (CIN) [150].

Like BC, lung cancer is also one of the most common diseases worldwide (2.09 million cases according to WHO data for 2018). One of the negative points of this cancer is the asymptomatology of the early stages. Therefore, the search for predictive markers that make it possible to identify the presence of cancer is important for a good prognosis. Chronic obstructive pulmonary disease (COPD) is a predisposing factor for this type of cancer. Deja et al. reported an increase in *N*-acetylated glycoproteins in patients with multi-stage lung cancer compared to COPD patients. They also pointed out that the *N*-acetylated glycoprotein signal is a useful marker for distinguishing between different stages of lung cancer [92].

Chandler et al. also showed a positive association between GlycA and incident colorectal cancer (CRC) and mortality by evaluating the baseline measurements of GlycA in two large cohorts—the Women’s Health Study (WHS) and the Multi-Ethnic Study of Atherosclerosis (MESA)—with a median follow-up period of 19 and 11 years, respectively [105].

Duprez et al. also pointed out that among other inflammatory markers such as high-sensitivity C-reactive protein (hsCRP), IL-6, and D-dimer, GlycA was the only one that was independently predictive of future cancer. It is curious to note the difference in importance in different ethnicities; GlycA did not predict total cancer in whites, but was a strong predictor in blacks, Chinese, and Hispanics [106].

However, other studies have shown a different trend: a relationship between low levels of NAG and the risk of developing hepatocellular carcinoma (HCC) [151] and urothelial carcinoma (UTUC) in patients compared with healthy controls [152]. In addition, another ^1^H-NMR metabolomic study in glioma, the most common of all primary central nervous system tumors, showed a lower level of glycoproteins, among other metabolites [153].

##### Metabolic Disorders

Obesity and diabetes are some of the most common metabolic disorders in which ^1^H-NMR glycoproteins have been studied. Both disorders are closely related to each other and to other pathologies such as cardiovascular diseases (CVDs) or metabolic syndrome (MetS). Systemic inflammation is hypothesized as a central mechanism.

##### Obesity

A higher body mass index (BMI) is characterized by higher leptin concentrations and decreased anti-inflammatory adiponectin levels. These parameters are determined with the leptin/adiponectin ratio, which is elevated in obesity and a marker of the adipose tissue production of pro-inflammatory cytokines and of insulin resistance in nondiabetic individuals [154,155]. An important result in terms of ^1^H-NMR glycoproteins and obesity is the considerable association found between GlycA and the leptin/adiponectin ratio [94,156], which suggests that GlycA is a marker of adipose tissue-associated low-grade inflammation.

Furthermore, GlycA has been shown to correlate with higher concentrations of triglycerides and other lipid levels, such as LDL cholesterol, in obese non-pregnant subjects [157] and in obese and overweight pregnant women [95]. The level of GlycA has also been shown to correlate with the amount of branched chain amino acids [157], which along with aromatic amino acids are increased in obesity and insulin resistance [96], and also in type 2 diabetes [158]. Lorenzo et al. also found a strong relationship between C-reactive protein (CRP), GlycA, and GlycB, and measures of insulin resistance and adiposity. Furthermore, GlycB has weaker relationships with CRP and measures of insulin resistance and adiposity than GlycA [97].

Strategies to reduce obesity such as an exercise-based lifestyle, diet, and even bariatric surgery have been the subject of countless studies. One way to establish whether ^1^H-NMR glycoproteins are directly related to poorer health in obese patients is to study whether they vary when these strategies are carried out. Barlett et al. evaluated how an exercise-based lifestyle or exercise plus diet interventions for 6 months modulate GlycA in sedentary adults with prediabetes. Their results showed a reduction in GlycA levels, which they associated with a decrease in visceral adiposity [136]. GlycA has also been measured in obese patients undergoing bariatric surgery, which is the most effective therapy in cases of severe obesity. The most typical forms of bariatric surgery are Roux-en-Y gastric bypass and sleeve gastrectomy, both of which lead to substantial weight loss. Manmadhan et al. saw that postoperative changes in GlycA were very positively associated with changes in body weight, high sensitive CRP (hsCRP), and glycated haemoglobin (HbA1c), and inversely with changes in the mean particle size of high-density lipoprotein (HDL) and adiponectin [159].

Few studies have been conducted in children or adolescents. One of them, a cohort of 1664 US adolescents from the HEALTHY study (risk factors for type 2 diabetes in a sixth-grade multiracial cohort), showed that high GlycA values were associated with higher BMI and more related to girls than to boys, which can be explained by the progression of puberty [160]. A second study in an adolescent population showed a significant reduction in GlycA after 12 weeks of lifestyle intervention consisting of weekly nutrition and health classes delivered by promoters (bilingual, bicultural health educators) and 3 days per week of moderate to vigorous physical activity. Decreases in GlycA were associated with decreases in 2-hour glucose (*p* < 0.008) and BMI (*p* < 0.03) [161].

##### Diabetes Mellitus 

Diabetes is a chronic disease that occurs either when the pancreas does not produce enough insulin (type 1 diabetes) or when the body cannot effectively use the insulin it produces (type 2 diabetes) [162]. Low-grade systemic inflammation has been associated with the risk of diabetes [163,164].

The association of circulating levels of inflammatory proteins, in particular APP, in type 2 diabetes (T2DM) is well described in prospective epidemiological studies [165,166,167]. One of the largest studies was conducted in 2012 by Würtz et al., who investigated the associations of circulating metabolites with fasting and post-loading glycemia before disease onset. They pointed out that 1α-acid glycoprotein is a predictor of future glycemia, and underlined the importance of prolonged inflammation as a risk marker for attenuating glucose tolerance [98]. In a set of 26,508 women enrolled in the WHS, Akinkuolie et al. showed the potential role of glycans in the risk of T2DM by showing that several APPs were associated with the risk of developing T2DM [101]. Connelly et al. confirmed the findings of Akinkuolie et al. [101] by showing that in 4525 participants of the Prevention of Renal and Vascular End-Stage Disease (PREVEND) study, GlycA was an independent predictor of T2DM even after adjusting for traditional diabetes risk factors and hsCRP [100]. Moreover, in PREVEND, the associations of GlycA with future T2DM were similar for men and women while the hsCRP associations appeared to be stronger in women than in men. Another study on patients with T2DM hospitalized for diseases such as congestive heart failure (CHF), cardiac non-CHF, infection, and other noncritical diseases showed that there were differences in inflammatory markers across disease states. GlycA was associated with higher IL-6 and CRP, with values being highest in T2DM patients with infectious diseases [99].

Lorenzo et al. studied the relation of GlycA, GlycB, and CRP with direct measures of insulin sensitivity. They found that CRP levels and GlycA were higher in T2DM than in isolated impaired glucose tolerance, but GlycB was not increased. They also found that GlycA had a more robust correlation with CRP, plasma glucose, and measures of adiposity and insulin resistance than GlycB [97]. In line with these results from Lorenzo et al., Fizelova et al. also found a positive association between GlycA and impaired insulin secretion in a population of 5401 non-diabetic men from the prospective Metabolic Syndrome in Men study (METSIM), in addition to the above-mentioned results by other authors with hyperglycemia, incident T2DM, and CVD [102].

In contrast to these results, some authors have found inverse relationships. Rawat et al. found that residual signals of *N*-acetyl glycoproteins were lower in diabetes patients with inadequate glycaemic control and with diabetic neuropathy, nephropathy, and cardiovascular disease than in healthy controls [168]. They attribute this to possible greater oxidative damage but more studies are needed to find a solid explanation. Likewise, Gruppen et al. found in one study that GlycA was not positively associated with T2DM [103]. However, in a similar study, they subsequently verified that GlycA was higher in subjects with either T2DM, MetS, or both [140].

##### Metabolic Syndrome (MetS)

Metabolic syndrome (MetS) is a clustering of cardiovascular risk factors with insulin resistance as a major feature. This syndrome has been defined in various ways, but generally consists of three or more of the following components: hyperglycemia, hypertension, hypertriglyceridemia, low HDL, increased waist circumference, and/or high BMI [169].

Pro-inflammatory markers such as white blood cell counts and plasma levels of coagulation factors (fibrinogen and plasminogen activator inhibitor 1), APP such as CRP and serum amyloid A (SAA), pro-inflammatory cytokines (tumor necrosis factor (TNF)-a, IL-1b, and IL-6), and chemokines are positively correlated with insulin resistance and the features of the metabolic syndrome in most cases [170]. Little information is available on the relationships of MetS with other pro-inflammatory biomarkers, but increased levels of glycosylated APP (GlycA) have been associated with MetS and negatively related to bilirubin levels [156]. This may represent a quantitative measure of a pro-inflammatory state.

In line with these results, Gruppen et al. found a higher concentration of GlycA in MetS subjects and a positive correlation of GlycA with cholesterol acetyl transferase (LCAT), systolic blood pressure, BMI, waist circumference, and plasma triglycerides. The correlation with HDL cholesterol was inverse [140,171].

##### CVD Risk and All-Cause Mortality Prediction

In the clinical field, the hsCRP marker has been strongly associated with CVD [172,173], as have other widely used markers such as IL-6, which has been more strongly related to all-cause death than CRP [174]. Interestingly, in the last few years, several studies have pointed to ^1^H-NMR glycoproteins, specifically GlycA, as better predictors of the risk of CVD and all-cause mortality in both the general population and the population at high risk of CVD [100,101,105,106,107,108].

##### Apparently Healthy Populations

GlycA has been measured in apparently healthy individuals from large population studies such as WHS, ‘Justification for the Use of Statins in Primary Prevention: An Intervention Trial Evaluating Rosuvastatin’ (JUPITER) trial, and MESA. In all of them, elevated baseline circulating glycoprotein *N*-acetyl methyl groups were associated with longitudinal risk of CVD incidence and mortality, among other pathologies such as chronic inflammatory-related severe hospitalization, cancer, or death [106,108,175,176]. Considering the results, the authors defend GlycA’s role as an important predictor of 5 to 15-year risk of CVD [107,108]. In these studies, the predictive capacity of GlycA was independent of age, sex, modifiable lifestyle risk factors, medication, and disease prevalence.

A large study conducted by Ritchie et al. in 2015 focused on GlycA levels in a population of 11,825 individuals from three different cohorts; i) the Dietary, Lifestyle, and Genetic Determinants of Obesity and Metabolic syndrome (DILGOM) study; ii) a large Finnish population survey on risk factors on chronic, noncommunicable diseases (FINRISK); iii) and the Cardiovascular Risk in Young Finns Study (YFS). The results suggested that in apparently healthy individuals, GlycA may be chronically elevated for periods of up to a decade; in individuals with elevated GlycA, cytokines also increased slightly, which suggested a prolonged low-grade inflammatory state. GlycA strongly predicted the future risk of hospitalization and death from infection [80].

Benson et al. wanted to take one step further and, in 6479 MESA participants, demonstrated that lower levels of GlycA were related to cardiovascular health (CVH) as defined by Life’s simple 7 score (LS7), which includes seven individual health metrics (smoking, physical activity, BMI, diet, total cholesterol, blood pressure, and blood glucose). LS7 scores were categorized into CVH groups classified as “optimal” (12–14, “average” (8–11), or “inadequate” (0–7). GlycA was independently inversely associated with continuous LS7 scores [176].

##### Prediction of CVD in High-Risk Populations

Recently, glycoprotein acetyls have been strongly related with myocardial infarction (MI), ischemic stroke (IS), and intracerebral hemorrhage (ICH) [177]. GlycA and its role in improving the prediction of CVD risk in high-risk populations has also been described [178].

Some studies carried out on patients from the angiography registry of the Intermountain Heart Study demonstrated that baseline levels of both GlycA and GlycB were strongly associated with future major adverse cardiovascular events [109]. In addition, GlycA proved to be an independent predictor of cardiac death [111]. Muhlestein et al. studied 2996 patients undergoing angiography for coronary artery disease (CAD) from the same trial, and determined that the interaction between GlycA and hsCRP was statistically significant for the outcome of death [112].

On another note, Correia et al. evaluated changes in key metabolites in 28 children undergoing surgery for congenital heart. A relationship was found between inflammation and metabolic derangement in the days after surgery for congenital heart disease with an increase in *N*-acetylated glycoprotein fragments immediately post-surgery [179].

##### Life Expectancy Prediction and All-Cause Mortality

In line with these results, McGarrah et al. studied 7617 cardiac catheterization patients from the CATHeterization GENetics (CATHGEN) biorepository. They also found a strong association of GlycA with mortality, CAD and all-cause mortality, cardiovascular and non-cardiovascular. They noted that the individuals at highest risk of dying were those who had diabetes and a higher concentration of GlycA, followed by those without diabetes but with high concentrations of GlycA, and finally individuals with diabetes but lower concentrations of GlycA. On the other hand, the GlycA and smaller HDL subclasses had independent but opposite effects on mortality risk prediction, with smaller HDL subclasses being protective [113].

A sub-study analysis of the trial Atherothrombosis Intervention in Metabolic Syndrome with Low HDL/High Triglycerides and Impact on Global Health Outcomes (AIM-HIGH) conducted by Otvos et al. confirmed and extended the previous findings. GlycA predicted CVD events and mortality in high-risk patients with established CVD who had achieved very low LDL-C levels. All-cause mortality was significantly associated with both GlycA and low levels of small HDL particles [114].

The remaining life expectancy is an increasingly used measure of survival. Gruppen et al. determined that men and women from the PREVEND study with higher GlycA levels had a lower life expectancy, while high levels of hsCRP were only related to a lower life expectancy in men [115].

##### Human Immunodeficiency Virus (HIV)-Infection

As far as we know, there is only one study in the literature that relates GlycA to HIV infection. Tibuakuu et al. investigated the association of GlycA with CVD risk in HIV infection, since in these patients the risk of CVD is higher than in people who are not infected [180,181]. They showed that GlycA levels were higher in men who were HIV-infected than in those who were not, and higher in men with detectable versus undetectable viral load. In HIV men with plaque, GlycA was positively associated with the extent of coronary artery calcium and total plaque [181]. Further research is needed to see if GlycA levels are predictive of incident CVD events in HIV-infected individuals.

Another study measured GlycA in HIV patients, but it focused on observing the effects of antiretroviral treatment [138] and will be discussed below.

##### Chronic Inflammatory Diseases Related to the Immune System

##### Rheumatoid Arthritis (RA)

RA is a chronic inflammatory disease associated with the development of CVD. GlycA may be a useful marker of disease activity and CVD risk in patients with RA. It has been shown that GlycA is higher in RA patients than in controls [83,118,119] and strongly correlated with all components of DAS28 scores, the measure of disease activity in RA [119]. Fuertes-Martin et al. have pointed out that the parameters H/W GlycA and GlycB ratios are significantly higher in the RA population than in controls and have hypothesized that the high, narrow shape of the peaks could be an additional marker of systemic inflammation [83].

Moreover, GlycA has been associated with the presence of coronary artery calcium and prevalent coronary artery disease in patients with RA [119] even though GlycA is predominantly associated with typical systemic inflammation and less with adiposity [118].

Bearing in mind that some traditional markers used to evaluate RA, such as CRP and ESR, are nonspecific because their concentrations are also increased in other chronic inflammatory diseases, GlycA could be an additional measure of inflammation that will lead to greater accuracy than if only the classic inflammatory parameters are considered, as is conventional in clinical practice [83,118].

##### Systemic Lupus Erythematosus (SLE)

Some studies have shown a high association between increased GlycA levels and SLE [120,121,122]. Two in particular have demonstrated that GlycA levels increased with each unit increase in the Systemic Lupus Erythematosus Disease Activity Index (SELDAI) [120,121].

In addition, GlycA has been shown to be a good marker of systemic inflammation in lupus nephritis, one of the most severe complications of SLE. Unlike GlycA levels, the CRP concentrations of non-lupus nephritic controls were not significantly different from those of patients with active SLE. It is important to stress the role of GlycA and BMI in predicting proliferative status over classical inflammation markers [122].

##### Psoriasis

Psoriasis is a chronic inflammatory skin condition associated with chronic systemic inflammation, increased vascular inflammation and a greater risk of incident CV events and CV mortality [182,183]. GlycA was seen to be increased in psoriasis patients and remained significant after adjustment for age, sex, BMI, and traditional CV risk factors. Interestingly, treatment of psoriasis with anti-TNF therapy led to a decrease in GlycA levels and vascular inflammation [123].

##### Inflammatory Bowel Disease (IBD)

IBD is a global disease that is increasingly prevalent on all the continents [184]. It is characterized by chronic relapsing intestinal inflammation [185]. Dierckx et al. measured GlycA in populations with ulcerative colitis (UC) and Chron’s disease (CD). GlycA reflected the inflammatory status of patients versus controls and the decrease in inflammation in response to treatment better than other classical markers such as PCR and fecal calprotectin (fcal) [124].

##### Other Chronic Inflammatory Diseases

COPD is a heterogeneous condition with patients displaying varying clinical and pathophysiological features. The mechanisms and mediators underlying COPD and its comorbidities are poorly understood. However, there is compelling evidence to suggest that increased oxidative stress and lung inflammation play an important role in its pathophysiology [186].

In an 8-year follow-up study, Ritchie et al. found a significant positive association between GlycA’s constituent glycoprotein α1-antitrypsin and increased risk of liver diseases, heart failure, and COPD and a significant association between GlycA’s constituent glycoprotein α1-acid glycoprotein and heart failure and chronic lower respiratory diseases [85]. Kettunen et al. made a systematic evaluation of GlycA as a reproducible biomarker for disease prediction in a population of 11,861 adults. Their results were the same as some of those mentioned so far and also demonstrated new strong and consistent associations between elevated GlycA and increased risk of alcoholic liver disease, chronic renal failure, glomerular diseases, COPD, inflammatory polyarthropathies, and hypertension [116].

As a curiosity, the relation is mentioned in two traditional Chinese medicine studies that detected lower plasma glycoprotein concentrations in patients with COPD and abnormal Savda syndrome than in controls [187], the Savda syndrome being a set of psychological and emotional stressors [188]. More rigorous studies and Western medicine are needed to establish a scientific hypothesis on the role of ^1^H-NMR glycoproteins in COPD.

Another chronic low-grade inflammation disease is chronic kidney disease (CKD), characterized by a reduced estimated glomerular filtration rate (eGFR) and/or albuminuria. However, some epidemiological studies have reported contradictory data that support a relationship between CKD and an increase in CRP [189], and others have not found any such association with CRP but with other inflammatory markers such as TNF-α and IL-6 [190,191].

Titan et al. investigated the association of GlycA to albuminuria and eGFR in 5050 middle-aged men and women from the Brazilian Longitudinal Study of Adult Health (ELSA-Brasil Study). They showed that GlycA was independently associated with albuminuria and inversely related to eGFR. They also showed that GlycA was better than hsCRP at diagnosing albuminuria, which suggests that glycation has an important role in the progression of CKD and in risk assessment [125].

Finally, chronic hepatitis C (CHC) has been most widely studied in the context of non-invasive biomarkers. Increased severity of fibrosis has been associated with higher NAC plasma levels [126].

##### Cognitive Function and Psychological Health

Psychological suboptimal health is a prevalent state with a pathophysiological mechanism that is extremely complicated and poorly understood but inflammation is known to be related. CRP and IL-6 have been associated with cognition, but few studies have measured inflammatory markers as predictors of cognitive function in middle age or of the onset of cognitive complications such as dementia [127]. However, a higher level of *N*-acetyl-glycoproteins in patients with psychological suboptimal health has been reported [192]. Another study showed an inverse relationship between GlycA and global cognition and also between information processing speed and memory domains [127].

One of the most studied cognitive diseases with the most mysterious aetiology is Alzheimer’s disease (AD). The need for early diagnosis is a growing issue today. MetS and elevated circulating glycoproteins were also associated to risk of AD and mild cognitive impairment (MCI) [128]. These results suggest that bringing these factors together would be more conducive to developing AD but further research is needed.

The lack of biomarkers of inflammation in this field and the results discussed above make the search for prediction and prevention biomarkers an increasingly attractive field.

#### 5.2.2. Rare Vascular Diseases

##### Takayasu Arteritis (TA)

TA is a rare, idiopathic systemic inflammatory disease affecting large arteries, including the aorta, its major branches, and the pulmonary arteries. Arterial inflammation is the core feature of the disease, variably associated with a systemic acute-phase response [193]. Novel biomarkers are required to distinguish inflammatory and non-inflammatory remodeling in Takayasu arteritis. It has been shown that *N*-Acetyl glycoproteins are significantly up-regulated in TA patients [130].

A larger study, conducted by Jain et al. in another cohort, confirmed these results and confirmed the role of glutamate and NAG as potentially strong biomarkers of TA [129].

##### Kawasaki Disease

Kawasaki disease (KD) is a self-limited vasculitis that typically presents in young children as an acute illness with fever and mucocutaneous changes. It can develop coronary artery aneurysms, and predispose to serious long-term cardiovascular complications [194]. In a pediatric population with acute KD disease, high levels of GlycA were confirmed. It was also found that GlycA and lipoproteins, both measured with ^1^H-NMR, may be useful for distinguishing acute KD from bacterial or viral illnesses [110] but further research is needed.

##### Primary Aldosteronism (PA)

PA is characterized by the autonomous production of aldosterone, which causes sodium retention, plasma renin suppression, endocrine hypertension, and cardiovascular damage, among other things [195]. Aldosterone is associated with key functions in the regulation of blood pressure, but has also been associated with causing inflammation, fibrosis, and blood vessel remodeling [196]. Berends et al. found that GlycA levels were significantly higher in a PA population than in normotensive control subjects and subjects with treated and untreated hypertension, which indicated enhanced low grade chronic inflammation [131].

##### Sickle Cell Disease (SCD)

SCD is a multisystem disorder with multiple organ damage associated with recurring inflammation caused by tissue ischemia, reperfusion injury, and vascular damage [197]. Although inflammatory markers used in clinics such as interleukins, prostaglandin-E2, tumour necrosis factor-α, and CRP are increased in SCD, GlycA is not. This result goes against what is reflected in this review and has been attributed to the fact that hemolysis is observed in SCD patients but not in patients with other pathologies. It should also be noted that haptoglobin is one of the major proteins in the GlycA signal and in this case, it depletes rapidly during intravascular hemolysis [132].

##### Human African Trypanosomiasis (HAT)

Very few studies on metabolomic profiling use ^1^H-NMR to identify a metabolic signature of a specific parasitic infection. Marked differences have been shown in plasma HAT patients who have a significant increase of creatinine, *N*-acetyl glycoprotein (*p* < 0.01), formate, and myoinositol compared to controls [133].

#### 5.2.3. Treatment Effects and Lifestyle

##### Tobacco Smoking

Tobacco smoking is one of the major preventable causes of death by CVD and cancer in the world. Higher levels of some inflammatory markers are indicators of exposure to smoking. Kianoush et al. studied the association between smoking and systemic inflammation (GlycA) in 11,509 participants from MESA and ‘The Brazilian Longitudinal Study of Adult Health’ (ELSA-Brasil) cohorts. They found similar significant associations between different measures of smoking behavior and higher GlycA and hsCRP levels [134].

##### Effect of Exercise

Increased physical activity and weight loss are effective ways to reduce inflammation. Only a few studies have described how these lifestyle changes contribute to reducing GlycA [98,135]. One study mentioned above with a 6-month intervention of resistance exercise alone or combined with diet in overweight and pre-diabetes individuals showed that GlycA levels decreased significantly by 2% [136]. Barber et al. demonstrated significantly reduced plasma GlycA in 1568 individuals submitted to 14 exercise interventions even after adjustment for age, sex, race, baseline BMI, and baseline GlycA [137].

A very interesting study by Kujala et al. shows clear differences between physically active and physically inactive age- and sex-matched pairs and twin pairs. They found better metabolic health including a decrease in isoleucine, α1-acid glycoprotein, and glucose in the physically active subjects [135].

##### Effect of Treatments

In clinical terms there is a need to be able to measure the effect of pharmacological treatments with reliable markers. GlycA has been measured in a few drug studies to evaluate its anti-inflammatory effect and in the near future it could be a clinically relevant biomarker for monitoring disease severity.

##### Modulators of Inflammatory and Immune Response

Anti- TNFα therapy has been established as an efficacious therapeutic strategy in inflammatory diseases. Interestingly, the treatment of psoriasis with anti-TNFα therapy led to a decrease in GlycA levels and vascular inflammation in close parallel with reductions in atherosclerotic CVD activity [123].

The effect of some monoclonal antibodies on GlycA levels has also been reported in the literature. Dierckx et al. showed a consistent decrease in GlycA levels in IBD patients during therapy with adalimumab, infliximab, vedolizumab, and ustekinumab compared to other biomarkers such as fecal calprotectin or CRP [124], which have had a moderate response at most in some patients with IBD [198].

##### Antiretroviral Therapy

Kelesidis et al. investigated how markers of inflammation change in response to treatment in a longitudinal antiretroviral therapy study. They found that GlycA was the only marker of inflammation, above hsCRP, IL-6, and D-dimer, that decreased across all the treatment groups conducting an initial antiretroviral therapy with atazanavir, raltegravir, and darunavir [138]. Even though future studies are needed to determine the role of protein glycans in HIV-1 infection, this finding suggests that GlycA could be a marker for evaluating the success of treatment in these patients.

##### Statins

Statins inhibit a key step in the biosynthetic pathway of sterol by reducing cholesterol and contributing to the prevention of cardiovascular disease. Although statins decrease some inflammation markers such as CRP, they do not appear to affect GlycA levels. More studies are required to confirm this [114].

##### Metformin

Metformin is a widely prescribed medication that has been used to treat T2DM. NAC serum levels decrease in metformin-treated T2DM patients compared to untreated patients [139].

##### Probiotics

The use of probiotics is an emerging approach for reducing chronic inflammation, but few studies have evaluated the effect of probiotics on inflammatory markers. In the literature, greater gut microbiota richness is negatively linked with the inflammation marker GlycA [86] in overweight pregnant women. More studies are needed to confirm that probiotics can play a role in reducing GlycA levels.

#### 5.2.4. Other Conditions

##### Sodium Intake

High sodium intake has been linked to major health issues such as CVD and hypertension. In a large cohort of predominantly healthy men and women with age- and sex-adjusted analyses that took into account BMI, lower GlycA, and hsCRP concentrations were both associated with higher 24-h sodium excretion, and these relations remained present after other potential covariates were taken into account [140].

##### Pregnancy

Strategies increasingly focus on tracking metabolic changes during pregnancy in order to determine metabolic profiles that may be associated with prenatal disorders. ^1^H-NMR has been used to discover metabolic biomarkers that personalize the monitoring of the pregnancy [141], but only a few studies have included ^1^H-NMR glycoproteins.

An untargeted ^1^H-NMR study of maternal blood plasma has shown a gradual increase in *N*-acetyl glycoproteins and a direct link between them and LDLc+VLDLc [141]. Another study conducted in overweight pregnant women evaluated the association between intake of dietary nutrients and markers of low-grade inflammation. Multiple nutrients correlated with GlycA—including fibre, LC-PUFA and α-3 LC-PUFA and several vitamins and minerals—but no correlations were detected between any of the nutrients and hsCRP and lipopolysaccharide (LPS) [86]. Similarly, increased richness in intestinal microbiota was negatively related to GlycA, but no similar relationship was observed between hsCRP and microbiota-rich gut [86], which suggests that GlycA may have inflammatory pathways different from those of CRP. The same cohort was used to study whether intestinal permeability was related to metabolic risk markers. The results showed that serum zonulin, a protein responsible for regulating paracellular transport in the intestine, was associated with GlycA, among other markers [95].

Houttu et al. studied whether there were differences in the inflammatory profile of overweight pregnant women and obese pregnant women. In line with what has been mentioned so far, low-grade inflammatory markers, GlycA, and hsCRP were statistically significantly higher in obese pregnant women than in overweight pregnant women. The correlation coefficients were also higher between GlycA and lipids than between hsCRP and lipids. Houttu et al. also found a correlation between GlycA and branched chain aromatic amino acids in pregnant women, coincidental with higher insulin and glucose concentrations during early pregnancy [142].

##### Toxicity

Drug-induced hepatotoxicity is an important healthcare issue in the sense that side effects in patients can be serious. Currently, toxicity is only detected when the tissue has already been badly damaged [143]. For this reason, new markers are required to warn of the toxicity of drugs and to better monitor the treatment. Huo et al. used ^1^H-NMR to find that the *N*-acetyl moieties of glycoprotein were significantly increased (*p* < 0.01) in sodium valproate-induced hepatotoxicity in epileptic patients, among other metabolites such as glucose, lactate, acetoacetate, VLDLc/LDLc, lysophosphatidylcholines, phosphatidylcholines, choline, creatine, amino acids, pyruvate, and uric acid [143].

It should be noted that predicting the toxicity of radiotherapy treatment is also a new research avenue in oncology because of the temporary toxicity generated that seriously affects the patient’s quality of life. In a case of head and neck squamous cell carcinoma (HNSCC), the toxicity of the treatment was studied via ^1^H-NMR of human blood serum. The high acute radiation sequelae were associated with increased *N*-acetyl glycoprotein signals, concordant with a significant increase in CRP levels and suggestive of an elevated inflammatory state [144].

## 6. Conclusions

### 6.1. ^1^H-NMR Glycoproteins as a Diagnostic Tool

In this review we have discussed the clinical importance of glycoproteins in the onset of some diseases. Specifically, we have summarized the main results of the clinical studies carried out to date on plasma glycoprotein concentrations detected using ^1^H-NMR spectroscopy, since it is the main high-performance metabolomic technique capable of quantifying serum or plasma glycoproteins in a sensitive and robust way.

Although the role of glycans in disease mechanisms is still not fully understood, the plasma level of glycosylation has been associated with different diseases, most of which have a marked inflammatory component. As has been mentioned in Section 3, plasma glycoproteins belong to the family of APP, which increase in concentration when there are inflammatory processes. To a great extent, they could be considered to be disease diagnostic markers. It is important to note that the ^1^H-NMR technique is a quantitative technique that measures a global state of glycation, but it cannot identify exactly which proteins are involved. All results have in common an increase in GlycA levels—or circulating levels of *N*-acetyl glycoproteins—with respect to a control group. The variable GlycB has been shown to be associated to these parameters but less strongly. However, this has been reported in so few studies [83,97,109,159] that firm conclusions cannot be drawn until more studies are performed.

Special mention should be made of the association found between GlycA and CRP. Although CRP is the inflammatory biomarker that has been most studied since ancient times, it has been shown to be prone to fluctuations for a variety of reasons [199].

These associations raise some open questions. The first is that the two markers may follow distinct inflammatory pathways because some studies have reported different behavior between GlycA and hsCRP [159]. The second is that GlycA may integrate more multiple inflammatory pathways by capturing the global signal of several proteins and, therefore, better captures the degree of systemic inflammation.

However, regardless of their possibly distinct inflammatory origin, GlycA has clear advantages over CRP: While hsCRP needs to be measured several times on consecutive days to ensure the risk of CVD, GlycA needs only one measure. On the other hand, Otvos et al. in previous studies [84] ensures a high reliability of GlycA because its measures are similar in both serum and plasma samples, in fasting and non-fasting states, and also after short or long-term storage, having GlycA a lower intra-individual variability than hsCRP.

### 6.2. ^1^H-NMR Glycoproteins as a Therapeutic Tool

Although more research is needed on GlycA and pharmacological treatments, the results of the studies lead us to hypothesize that GlycA’s response to treatments may help to improve treatment follow-up and bring us closer to the concept of personalized medicine in the future.

### 6.3. Future Perspectives

Throughout this review, we have observed how interest in ^1^H-NMR glycoprotein research in the clinical field has been increasing from year to year. From the results, we can conclude that characterization of glycoproteins by ^1^H-NMR has two main advantages. First, the versatility of the ^1^H-NMR technique, which from a single spectrum gives the inflammatory information provided by the glycoprotein profile as well as other information provided by metabolites such as lipoproteins. Second, the applications reviewed here demonstrate that GlycA is potentially a key biomarker in a wide range of diseases.

Even though most of the results are consistent, it is necessary to point out some aspects in which we must move forward in the future. The first is that the mechanism of glycation in human disease is not yet fully understood because of the highly complicated structures of glycans and their mechanism of action. Therefore, more research is needed. Secondly, there is a lack of a consensus on the technique for quantifying glycoproteins by NMR spectroscopy. The techniques used by different laboratories should be validated and standardized. For a rigorous transfer to the clinic in the future, these aspects must be addressed so that these inflammatory markers can become part of conventional clinical practice.

List of abbreviations: OC (ovarian cancer), CSCC (cervical squamous cell carcinoma), BC (breast cancer), LC (lung cancer), CRC (colorectal cancer), GlcNAc (*N*-Acetyl glucosamine), TG (total triglycerides), CRP (C-reactive protein), IL-6 (interleukin-6), T2DM (type 2 diabetes mellitus), MetS (metabolic syndrome), CVD (cardiovascular disease), CVE (cardiovascular event), COPD (chronic obstructive pulmonary disease), RA (rheumatoid arthritis), SLE (systemic lupus erythematosus), SELDAI (Systemic Lupus Erythematosus Disease Activity Index), IBD (inflammatory bowel disease), CKD (chronic kidney disease), CHC (chronic hepatitis C), AD (Alzheimer disease), MCI (mild cognitive impairment), TA (Takayasu arteritis), KD (Kawasaki disease), PUFA (polyunsaturated fatty acids), PA (primary aldosterism), SCL (sickle cell disease).

## Figures and Tables

**Table 1:** Examples of serum protein glycation changes and their association with disease.

Glycoprotein	Glycation Change	Related Diseases	References
Alpha 1-Acid Glycoprotein (AGP)	Highly branched *N*-linked glycan	Cirrhosis and HCC, congenital disorders, RA, SLE	[35,36]
	Increased sialylation	Cancer	[37]
	Decreased sialylation	Cirrhosis and HCC	[35,38]
	Increased fucosylation	liver cancer	[39]
Alpha-Fetoprotein	Elevated bisecting *N*-acetylglucosamine, decrease in sialylation and increase in fucosylation	Cirrhosis, hepatitis and HCC	[40,41,42]
Alpha -1-antitrypsin (ATT)	Increased fucosylation alpha	Hepatitis C, HCC	[43,44]
	Decrease in branching, predominance of alpha 2–6 linked sialic acid and less alpha 2–3 linked sialic acid	Breast and ovarian cancer	[45]
	Oligosaccharide branching and increased sialic acid content	Acute general inflammation	[46]
	Increased glycan branching	RA	[47]
Transferrin	Increased branching and fucosylation of *N*-glycans. Increasing peripheral *N*-acetylglucosamine residues	Ovarian, breast and colon cancer, HCC, Cirrhosis, hepatitis	[48,49]
	Increased fucosylation and sialic acid-linked to galactose	Liver disease	[50]
Haptoglobin (Hp)	Increased fucose and *N*-acetylglucosamine	Alcoholic liver disease	[51]
	Increased fucosylation	Various types of cancer and RA	[52,53,54,55]
	*O*-glycans ad fucosylation	Prostate cancer	[56]
Inmunoglobulin G (IgG)	Decreased galactose	RA, SLE, IBD, ovarian cancer, prostate cancer	[57,58,59,60,61,62,63]
	Increased *N*-acetylglucosamine residues (controversy)	RA	[64,65]
Inmunoglobulin A (Ig A)	Reduced galactosylation of *O*-linked glycosylation	Nephropathy	[66,67]

HCC (hepatocellular carcinoma), SLE (systemic lupus erythematosus), RA (rheumatoid arthritis), IBD (inflammatory bowel disease)

**Table 2:** Summary of ^1^H-NMR glycoprotein’s clinical applications. (a) Diseases.

Clinical Study Topic		Main Findings	References
**Tumors and cancer**	OC, CSCC, BC, LC, CRC	Increased circulating *N*-acetyl glycoproteins levels and increased GlcNAc-branching of *N*-glycans.	[24,89,90,91,92,93]
**Metabolic diseases**	Obesity	Association between GlycA and the leptin/adiponectin ratio	[94]
		Correlation between GlycA and TG and lipids	[95]
		Correlation GlycA and branched chain amino acids	[96]
		Strong relationship of CRP, GlycA, and GlycB and insulin resistance	[97]
	Diabetes Mellitus	α1-acid glycoprotein as a predictor of future glycemia	[98]
		Associations of GlycA with higher IL-6 and CRP	[99]
		Associations of GlycA with future T2DM	[100,101]
		GlycA had a more robust correlation with CRP, plasma glucose, and measures of adiposity and insulin resistance than GlycB	[97,102]
	MetS	Increased levels of glycosylated acute-phase proteins (GlycA) associated with MetS	[94,103,104]
**Cardiovascular risk**	Healthy individuals	GlycA/alpha1-acid glycoproteins or baseline circulating glycoprotein *N*-acetyl methyl groups are associated with CVD and longitudinal risk of all-cause mortality.	[101,105,106,107,108,109,110]
	High-risk individuals	GlycA and GlycB strongly associated with future major adverse CVE	[109]
		GlycA and hsCRP was statistically significant for the outcome of death	[111,112]
		GlycA, and small and medium-size HDL particles proved to be independent predictors of cardiac death.	[113,114]
	Life expectancy	Higher GlycA levels had lower life expectancy.	[115]
	All-cause mortality	Positive association between α1-antitrypsin and increased risk of liver diseases, heart failure, and COPD, and significant association between α1-acid glycoprotein and heart failure and chronic lower respiratory diseases	[85]
		GlycA related to increased risk of alcoholic liver disease, chronic renal failure, glomerular diseases, COPD, inflammatory polyarthropathies, and hypertension	[116]
**HIV-infection**		Higher GlycA levels in HIV-infected patients	[117]
**Chronic inflammatory diseases**	RA	GlycA is higher in RA patients than in controls.	[83,118,119]
	SLE	GlycA levels increased with each unit increase in SELDAI.	[120,121]
		GlycA has been shown to be a good marker of systemic inflammation in lupus-nephritis.	[122]
	Psoriasis	GlycA is increased in psoriasis.	[123]
	IBD	GlycA in populations with ulcerative colitis and Chron’s disease better reflects inflammatory status than other classical markers.	[124]
	CKD	GlycA was independently associated with albuminuria and inversely related to eGFR.	[125]
	CHC	Increased severity of fibrosis has been associated with higher NAC plasma levels.	[126]
**Cognitive function and** **psychological health**	Global cognitive function	GlycA is inversely related to global cognition, information processing speed and memory domains.	[127]
	AD	Elevated circulating glycoproteins were associated with the risk for AD and MCI.	[128]
**Rare vascular diseases**	Takayasu arteritis	*N*-Acetyl glycoproteins are significantly up-regulated in TA patients	[129,130]
	Kawasaki disease	High levels of GlycA were confirmed in paediatric population with acute KD disease	[110]
**Primary aldosteronism**		GlycA levels significantly increased in PA population.	[131]
**Sickle cell disease**		GlycA levels are decreased in SCL.	[132]
**Human African** **Trypanosomiasis**		Significant increase of *N*-acetyl glycoprotein in HAT patients.	[133]

**Table 3:** Summary of ^1^H-NMR glycoprotein’s clinical applications. (b) Treatment effects and lifestyle.

Clicical Study Topic		Main Findings	References
**Tobacco smoking**		Similar significant associations between different measures of smoking behaviour and higher GlycA and hsCRP levels.	[134]
**Effect of exercise**		Regular exercise significantly reduced plasma GlycA.	[135,136,137]
**Effect of treatments**	Anti-TNF and monoclonal antibodies	Decrease in GlycA levels.	[122,123]
	Antirretroviral treatment	GlycA was the only marker of inflammation, among hsCRP, IL-6 and D-dimer, that decreased.	[138]
	Statins	Do not affect GlycA levels.	[114]
	Metformin	Lower NAC serum levels in T2DM patients treated in metformin than in untreated patients.	[139]
	Probiotics	Greater gut microbiota richness is negatively linked with low-grade inflammation marker GlycA.	[86]

**Table 4:** Summary of ^1^H-NMR glycoprotein’s clinical applications. (c) Other conditions.

Clinical Study Topic		Main Findings	References
**Sodium intake**		Lower GlycA and hsCRP concentrations were both associated with higher 24-h sodium excretion.	[140]
**Pregnancy**		Gradual increase in *N*-acetyl glycoproteins during pregnancy.	[141]
		Multiple nutrient intake correlates with GlycA including fibre, LC-PUFA and w-3 LC-PUFA and several vitamins and minerals.	[86]
		GlycA and hsCRP were statistically significantly higher in obese than in overweight pregnant women.	[142]
**Toxicity**	Sodium valproate	*N*-acetyl moieties of glycoprotein significantly increased (*p* < 0.01) in valproate sodium induced hepatotoxicity	[143]
	Oncologic toxicity	The high acute radiation sequelae were associated with increased signals of *N*-acetyl glycoproteins	[144]

OC (ovarian cancer), CSCC (cervical squamous cell carcinoma), BC (breast cancer), LC (lung cancer), CRC (colorectal cancer), GlcNAc (*N*-Acetyl glucosamine), TG (total triglycerides), CRP (C-reactive protein), IL-6 (interleukin-6), T2DM (type 2 diabetes mellitus), MetS (metabolic syndrome), CVD (cardiovascular disease), CVE (cardiovascular event), COPD (chronic obstructive pulmonary disease), RA (rheumatoid arthritis), SLE (systemic lupus erythematosus), SELDAI (Systemic Lupus Erythematosus Disease Activity Index), IBD (inflammatory bowel disease), CKD (chronic kidney disease), CHC (chronic hepatitis C), AD (Alzheimer disease), MCI (mild cognitive impairment), TA (Takayasu arteritis), KD (Kawasaki disease), PUFA (polyunsaturated fatty acids), PA (primary aldosterism), SCL (sickle cell disease).

## Supplementary Materials

The following are available online at https://www.mdpi.com/2077-0383/9/2/354/s1, Table S1: Research articles related to the glycoprotein determination through 1H-NMR, Table S1—2: Metabolic diseases, Table S1—3: Cardiovascular risk, Table S1—4: HIV infection, Table S1—5: Chronic inflammatory diseases, Table S1—6: Cognitive function and psychological health, Table S1—7: Rare vascular diseases, Table S1—8: Pregnancy, Table S1—9: Primary aldosteronism, Table S1—10: Sickle cell disease, Table S1—11: Human African Trypanosomiasis, Table S1—12: Sodium intake, Table S1—13: Tobacco smoking, Table S1—14: Effect of excercise, Table S1—15: Toxicity, Table S1—16: Others.
