# Supplementary material for: Human Serum/Plasma Glycoprotein Analysis by 1H-NMR, an Emerging Method of Inflammatory Assessment
Source: J Clin Med. 2020 Jan 27;9(2):354. doi: 10.3390/jcm9020354 (PMC7073769; doi:10.3390/jcm9020354)
Supplement: Supplementary file 1 [file jcm-09-00354-s001.pdf]

Supplementary file

Title: Human Serum/Plasma Glycoprotein Analysis by ¹H-NMR, an Emerging Method of Inflammatory Assessment

Rocío Fuertes-Martín [1], Xavier Correig [2,*], Joan-Carles Vallvé [3] and Núria Amigó [1]

[1] Biosfer Teslab SL, Metabolomics platform, Universitat Rovira i Virgili,IISPV, CIBERDEM, 43201-Spain; namigo@biosferteslab.com

[2] Metabolomics platform, Universitat Rovira i Virgili, IISPV, CIBERDEM, 43007-Spain; xavier.correig@urv.cat

[3] Unitat de Recerca en lipids i arteriosclerosi, Hospital Universitari Sant Joan de Reus; IISPV; CIBERDEM, 43201-Spain; jc.vallve@urv.cat

* Correspondence: xavier.correig@urv.cat; Tel.: +34-977-559-623

Contents

1S. Glycoprotein profiling methods 2
The method by Otvos et al. 2
The method by Ala-Korpela et al. 2
Other methodologies 3
Table 1S. Research articles related to the glycoprotein determination through ¹H-NMR 4
1S.1. Tumours and cancer 4
1S.2. Metabolic diseases 9
1S.3. Cardiovascular risk 15
1S.4. HIV infection 21
1S.5. Chronic inflammatory diseases 22
1S.6. Cognitive function and psychological health 27
1S.7. Rare vascular diseases 28
1S.8. Pregnancy 29
1S.9. Primary aldosteronism 30
1S.10. Sickle cell disease 31
1S.11. Human African Trypanosomiasis 31
1S.12. Sodium intake 32
1S.13. Tobacco smoking 32
1S.14. Effect of excercise 33
1S.15. Effect of treatments 34
1S.16. Toxicity 34
1S.17. Others 35
References 37

1S. Glycoprotein profiling methods

Considering that in NMR the integrated surface (area) of the absorption signal is proportional to the number of nuclei that pass from the fundamental state to the excited state, integrating isolated signals is the classical approach to estimating the concentration of glycoprotein acetyls. Many authors use this method of integration to quantify the signal.[1–10] However, this approach is very sensitive to baseline distortions and it is not recommended for overlapping peaks. The results in many cases are expressed in 'units of detected protons' with one unit corresponding to the area of the proton NMR signal detected on 1 mmol/l formate in 1 ml of serum.[2]

Most of the studies reported in the literature, especially since 2015, use the following two methodologies to analyse the ^{1}H-NMR of glycoproteins. This information is reflected in in **table 1S**.

The method by Otvos et al.

The experimental NMR spectroscopy used by Otvos et al. to quantify GlycA is exactly the same as the one used in the numerous clinical studies carried out to assay lipoprotein subclasses and lipids described above. Briefly, they performed a curve fitting method to quantify the particle concentration and mean particle size of various lipoprotein subclasses (*LipoProfile*®)[11] at Liposcience Inc (acquired by Labcorp in 2014). For glycoproteins, they acquired the serum spectra in the same way as NMR LipoProfile® test spectra but in a single block of 8 scans. The GlycA signal was quantified by non-negative linear least-squares deconvolution of the 1.86-2.07 spectral region using proprietary software and the same singular value decomposition computation used for NMR LipoProfile® analysis.[12,13] The deconvolution models include a library of allylic proton reference spectra from 57 isolated lipoprotein subclasses (20 HDL, 9 LDL, 28 VLDL/chylomicron) that were obtained for use in the NMR LipoProfile® deconvolution model, sets of slightly offset narrow Lorentzian signals to model the chemical shift microheterogeneity of glycoprotein GlcNAc methyl signals centered at 2.00 ppm and also at other downfield locations (~2.02-2.05 ppm), a plasma protein reference spectrum background from amino acid residues on albumin and other plasma proteins. Summing the derived amplitudes of the 10 Lorentzian components between 1.99 and 2.01 ppm gives the sample's GlycA signal amplitude (US 2013/0328561 A1 patent). They applied a correction factor of 17.8 μmol/l of glycoprotein N-acetyl methyl group concentration units.[14] They evaluated the contribution of the acute phase proteins 1-antitrypsin, haptoglobin, transferrin, fibrinogen, IgG 1-acid glycoprotein, 1-antichymotrypsin, and 2-macroglobulin. Their results indicated that from these proteins, IgG, fibrinogen and 2-macroglobulin gave rise to no detectable GlycA NMR signal, while the other five acute-phase glycoproteins appear to have mobile glycan chains that would produce GlycA signals in proportion to their glycan GlcNAc concentrations.[14]

The method by Ala-Korpela et al.

In 2014, the Finnish company Brainshake (known as Nightingale Health Ltd since 2017) became a serum NMR metabolomics platform that measures more than 200 metabolites, including glycoprotein acetyls, in a highly automated way.[15] The experimental protocols are based on the lipoprotein profile characterization developed by Ala-Korpela, P. Soininen and colleagues in which they set up a curve fitting model by using Lorentzian functions with an in-house algorithm for deconvoluting the signals that mathematically optimized the half-line width, the resonance frequency and the intensity for each Lorentzian function.[16] Their NMR metabolomics platform includes three molecular windows for analysing the NMR spectrum: the LIPO window, the LMWM window, and the LIPID window. The LIPO window uses a Bruker NOESY solvent pre-saturation pulse sequence to analyse the largest molecules, and lineshape fitting and regression methods to quantify the number of particles in each lipoprotein subclass

and their content (mainly cholesterol and triglycerides). The LMWM window uses a Bruker 1D CPMG pulse sequence to analyse low molecular weight metabolites. And the LIPID window uses lipid extraction procedures to provide information about saturated and unsaturated fatty acid families, free and esterified cholesterol, sphingolipids and phosphoglycerides.[17] The peak of the glycoprotein acetyls is seen in the LIPO and LMWM window. For each metabolite a ridge regression model is applied for quantification to overcome the problems of heavily overlapping spectral data.[18] Low-molecular-weight metabolites and lipid extract measures are quantified in mmol/L using regression modelling calibrated against a set of manually fitted metabolite measures. The calibration data is quantified using iterative lineshape fitting analyses and PERCH NMR software (PERCH Solutions Ltd., Kuopio, Finland). Absolute quantification cannot be directly established for the lipid extract measures because of experimental variation in the lipid extraction protocol. [18]

Other methodologies

In 1987 Bell et al. were the first to assign the broad peaks centred at 2.04 ppm and 2.08 ppm to N-acetyl protons of N-acetylated carbohydrate side-chains associated with APP. They used a Bruker AM500 spectrometer operating at 500 MHz. To estimate the concentration of acute-phase glycoproteins in blood plasma by NMR, standard additions were made of a mixture of -acid glycoprotein, -antitrypsin, haptoglobin and transferrin. Peak intensities were compared by weighing paper traces. They suggested that the concentration of glycoproteins responsible for the two signals (I, 2.04 ppm and II, 2.08 ppm) was 13 mg/ml in the plasma of normal subjects.[19]

De Meyer et al. implemented the adaptive intelligent (AI)-Binning algorithm.[20] Briefly, each point in the NMR spectral range of a specific bin is evaluated as a potential (candidate) new bin edge. This candidate bin edge virtually splits the current bin into two new bins, and the quality of the two new bins is compared with the quality of the current bin. In order to assess this bin quality, a measure known as bin value is defined. The detection of the -1 acid glycoprotein peaks was more straightforward with AI-Binning than with standard binning. No arbitrary parameters, reference spectra, a priori knowledge or data modifications are required for this protocol.[20] Correia G. et al. used the R package BATMAN (Bayesian AuTomated Metabolite Analyser for NMR spectra) for deconvolution and relative metabolite quantification in the spectra.[21] Lecuyer et al. used another integrative methodology, NMRPipe, to slice each 1D NMR spectrum into buckets of 0.001 ppm, containing NMR signals.[22] The intelligent buckets were scaled to the total summed integrals for each spectrum and then used as the final input for the multivariable models.[23]

Finally, many authors use the entire normalized spectrum to make statistical analyses with SIMCA,[24–26] Orthogonal Projections to Latent Structures (O-PLS),[27–29] Partial Least Squares-discriminant Analysis (PLS-DA),[30,31] or Statistical Total Correlation Spectroscopy[32] (STOQSY).[33]

It should be noted that most of the studies described in this review use the methodology of Otvos (LipoScience-Labcorp) and Ala-Korpela, Soininen et al. (Brainshake-Nightingale). Both methodologies provide the parameter GlycA as the absolute N-acetyl group concentration. As far as we know, no comparative study of the two methods has been published. However, to our knowledge, the first study in which GlycA data analysed with Vantera, a clinical NMR analyzer designed for clinical use, have been reported is the PREVENT study. A high correlation (a coefficient of 0.983) was reported between the Vantera analyser from LiposScience (Labcorp) and Brainshake (Nightingale Health Ltd) GlycA analysis methods.[34] The studies in which other methodologies are used are not comparable with these two methods. Therefore, the N-acetyl NMR information needs to be unified in a single inflammatory marker such as GlycA. It would also be interesting to perform a comparative study of the results of the methodologies mentioned to quantify the N-acetyl groups of glycoproteins so that the studies can be comparable between them.

Table 1S. Research articles related to the glycoprotein determination through ^{1}H-NMR

1S.1. Tumours and cancer					
**Main objective**	Participants	Key methods	Main results related to glycoproteins	Clinical topic	Reference
**To characterize N-acetyl protons of highly mobile N-acetylated carbohydrate side-chains associated with 'acute-phase' plasma glycoproteins.**	> 10 healthy adults, 6 MG, 1 melanoma and 5 RA and > 10 pairs from bothmother and cord at birth	Bruker AM500 spectrometer<br>Standard additions of glycoproteins (spiking)<br>Plasma samples -20ºC storage	The intensities of peaks in the spectrum of maternal plasma are greater than those of the cord plasma. Peaks are also more intense in maternal plasma than in plasma from non-pregnant women. The intensities of peaks in the spectra of plasma from subjects with melanoma and RA are greater than those of normal plasma.	Pregnancy, melanoma and RA	Bell 1987[19]
**To study the variations in NAG and NANA glycosylated residues in three clinical situations: cancerous pathologies, acute inflammatory processes and autologous bone marrow transplantation (BMT).**	225 patients<br>49 controls	Bruker AM 400 MHz spectrometer<br>Plasma samples -20ºC	i) The distribution of glycosylated residues varies with the origin of the cancerous tissue;<br>ii) The level of these residues is a function of tumour development;<br>iii) The concentrations in NAG and NANA are well correlated with the standard biological parameters of acute phase and leucocyte activation.	Cancerous pathologies	Kriat et al. 1991[1]
**An unassigned and prominent resonance in the region from d 2.0–2.1ppm has frequently been found in the in vivo MR spectra of cancer patients. 1H-NMRS on the aspirated cyst fluid (in vitro) of patients confirmed the observation.**	11 Ovarian tumour patients	Bruker DMX-500 spectrometer<br>Cyst fluid stored at -70ºC<br>COSY	N-acetyl groups from glycoproteins and/or glycolipids may contribute to the d 2.0–2.1ppm<br>resonance complex in ovarian cyst fluid.	Ovarian tumour	Kolwijck et al 2009[4]

**To examine the association of baseline GlycA concentration with total death, incident CVD, chronic inflammatory-related non-CVD, ChrIRD events and total cancer, and to compare these associations with other commonly used clinical biomarkers of chronic inflammation (hsCRP, IL-6, and D-dimer).**	6523 men and women from MESA (healthy at baseline). Median follow-up 12.1 y.	NMR-algorithm at LabCorp, Inc. (formerly LipoScience)[14] Plasma samples	Relative risk per SD of GlycA, IL-6, and D-dimer for total death; for total CVD; and for ChrIRD. Only GlycA was predictive for total cancer. Women had 7% higher values of all inflammatory biomarkers than men and a significantly lower GlycA prediction coefficient than men in predicting total cancer.	All-cause mortality (CVD, ChrIRD, cancer)	Duprez et al.2016[35]
**To reveal the changes in the metabolic profiles during BC progression. Detection of early stage and late stage altered metabolic pattern.**	72 early- and late-stage BC patients 50 healthy subjects	Bruker Avance III 800 MHz NMR spectrometer CPMG pulse Plasma samples	The levels of hydroxybutyrate, lysine, glutamate, glucose, NAG and lactate were highly distinguished in BC. ROC curve showed that glutamate, lactate and NAG levels of metabolic alterations separate EBC and LBC with an AUC value of 0.7	Breast cancer: EBC and LBC	Suman et al.2018[31]
**To investigate whether metabolomic profiles, generated from a simple blood draw from healthy women, could help predict the risk of developing breast cancer in the 10–15 subsequent years.**	206 breast cancer cases diagnosed during a 13-year follow-up 396 matched controls SU.VI.MAX cohort	Bruker AVANCE III 500 MHz NMR spectrometer NOESY1D and CPMG pulses Plasma samples	Women characterized by higher fasting plasma levels of valine, lysine, arginine, glutamine, creatine, creatinine and glucose, and lower plasma levels of lipoproteins, lipids, glycoproteins, acetone, glycerol-derived compounds and unsaturated lipids had a higher risk of developing breast cancer.	Risk of breast cancer developm ent	Lecuyer et al. 2018[23]

**To investigate the metabolic signatures of a glioma in plasma in order to assess the diagnostic potential of this approach and gain novel insights into the metabolism of glioma and its systemic effects.**	70 glioma patients<br>70 controls	Varian Unity Inova 600 spectrometer<br>CPMG pulse<br>Plasma samples	Patients with a glioma were associated with lower concentrations of isoleucine, leucine, valine, lactate, alanine, **glycoprotein**, glutamate, citrate, creatine, myo-inositol, choline, tyrosine, phenylalanine, 1-methylhistidine, α-glucose, β-glucose, and higher concentrations of very low-density lipoprotein, low density lipoprotein (LDL), unsaturated lipids, and pyruvate.	Glioma	Kelimu et al.2016[36]
**To examine the association between GlycA and incident CRC and mortality**	Discovery cohort: 27,495 participants from the WHS study (median follow-up 19 y.); Replication cohort: 6,784 participants from the MESA study (median follow-up 11 y.)	NMR-algorithm at LabCorp, Inc. (formerly LipoScience)[14]<br>Plasma samples	In WHS, adjusted HRs per SD increment of GlycA for CRC incidence and mortality were 1.19 (1.06±1.35; p = **0.004**) and 1.24 (1.00±1.55; p = **0.05**), respectively. Replicated findings in MESA showed that HRs per SD of GlycA for CRC incidence and mortality were 1.32 (1.06±1.65; p = **0.01**) and 1.54 (1.06±2.23; p = **0.02**), respectively, after<br>adjusting for age, sex, and race.	CRC	Chandler et al.2016[37]
**To investigate whether metabolic differences could be detected between HCC cases and matched controls from a prospective cohort study using serum samples collected prior to diagnosis.**	114 primary HCC cases<br>222 controls	Bruker Avance III 800 MHz spectrometer<br>CPMG and NOESY pulses<br>Serum samples	Sixteen metabolites of either endogenous or exogenous origin including lower levels of N-acetyl glycoproteins were found to be significantly associated with HCC risk.	Hepatocellular carcinoma	Fages et al. 2015[27]

**1H NMR-based metabolomic analysis of serum samples from patients with UTUC.**	39 UTUC patients<br>34 healthy controls	Bruker AV 500 MHz spectrometer<br>CPMG pulse<br>Serum samples	Serum LDL, VLDL, valine and glycoprotein levels followed a decreasing trend, whereas serum PUFA and 3,7-dimethyluric acid levels showed an increasing trend in UTUC patients compared with healthy controls.	UTUC	Li et al.2015[9]
**To use 1H-NMR to distinguish between the metabolic fingerprints of COPD and lung cancer patients**	77 NSCLC<br>22 COPD	Bruker Avance II 600MHz<br>CPMG<br>Plasma samples stored at -80ºC	Increased N-acetylated glycoproteins were observed in all lung cancer patients compared with the COPD group. These metabolite biomarkers may prove useful in distinguishing lung cancer states: isoleucine, acetoacetate, and creatine as well as the two NMR signals of N-acetylated glycoproteins and glycerol.	Lung cancer and COPD	Deja et al.2014[25]
**To investigate molecular processes that reflect acute radiation sequelae in HNSCC patients using NMR-based metabolomics of blood serum.**	45 patients with HNSCC:<br>low ARS (26 patients), high ARS (19 patients)	Bruker 400.13 MHz<br>Avance III spectrometer<br>NOESY, CPMG, DIFF, JRES pulses<br>Serum samples	The high ARS group was characterized by the increased signals arising from **NAG** and acetate as well as the decreased signals of branched amino acids, alanine, creatinine, choline containing compounds and carnitine.<br>Serum glucose is low in the high ARS group.<br>NAG is positively correlated with CRP, platelet count (PLT), ESR and absolute monocyte count and is also negatively correlated with albumin and mean platelet volume	Acute radiation sequelae in HNSCC	Boguszewicz et al. 2016[10]

**To carry out a 1H-NMR-based metabolic phenotyping study to identify coordinated metabolic serum changes associated with advanced metastatic breast cancer (MBC) in comparison to the localized early disease (EBC).**	46 EBC<br>39 MBC<br>Validation: 61 EBC and 51 MBC	Bruker Avance III spectrometer 800MHz<br>Standard 1H 1D NMR pulse sequences, NOESY and CPMG<br>Serum samples	9 statistically significant differences between EBC and MBC patients: histidine, acetoacetate, glycerol, pyruvate, glycoproteins (NAC1 $p<0.027$ and NAC2 $p<0.007$), mannose, glutamate and phenylalanine	Breast cancer: EBC and MBC	Jobard et al. 2014[24]
**To find a possible correlation between the t biochemical serum contents of a1-GP, a1-AT, Hp, CRP, IgA, IgM, IgG and Tf) and MRS data (NAG and NANA) in selected pathologies**	40 patients (cancer, inflammatory and infectious diseases, diabetes)<br>10 controls	Bruker AM 400-WB spectrometer<br>Serum samples<br>-80ºC storage	High correlation between MRS data and the most abundant acute-phase glycoproteins a1-GP, a1-AT and Hp. No correlation with Ig levels. Biochemical and MRs variables are independently sensitive to the inflammatory status of the patient. However, the combination of the two sets of variables fails to provide additional sensitivity and specificity.	Cancer, inflammat ory and infectious diseases, diabetes	Torri 1999[2]
**To use 1H NMR-based metabonomics to investigate esophageal cancer metabolic signatures in plasma and urine**	108 EC patients<br>40 healthy subjects	Varian Unity Inova 600MHz NMR spectrometer<br>CPMG, COSY, TOCSY<br>Plasma samples	Compared to controls, EC plasma had higher levels of dimethylamine, a-glucose, b-glucose, citric acid, and lower levels of leucine, alanine, isoleucine, valine, glycoprotein, lactate, acetone, acetate, choline, isobutyrate, unsaturated lipids, VLDL, LDL, 1-methylhistidine	EC	Hasim et al. 2012[8]

**To use 1H-NMR based metabonomics to characterize the metabolic profiles of cervical intraepithelial neoplasia (CIN) and cervical squamous cell carcinoma (CSCC).**	38 patients with CIN<br>38 patients with CSCC<br>38 healthy women	Varian Inova 600MHz spectrometer<br>CPMG<br>Plasma samples stored at -80˚C	Compared with samples from patients with CIN, the plasma of CSCC patients had higher levels of acetate, formate, lactate, isoleucine, leucine, valine, alanine, glutamine, histidine, tyrosine, acetylcysteine, myo-inositol, glycoprotein, α-glucose and β-glucose, together with lower levels of acetone, unsaturated lipid and carnitine.	Cervical carcinoma: CIN/CSCC	Hasim et al. 2013[7]

1S.2. Metabolic diseases					
**Main objective**	Participants	Key methods	Main results related to glycoproteins	Clinical topic	Reference
**To examine changes in GlycA after lifestyle intervention among young, obese, prediabetic Latinos.**	27 obese, prediabetic young Latinos<br>12-week lifestyle intervention	NMR-algorithm at LabCorp, Inc. (formerly LipoScience)[14]<br>Plasma samples	GlycA was significantly reduced (p<0.01)<br>Additional improvements were observed in multiple cardiovascular risk factors, including BMI, total cholesterol and 2-hour glucose.<br>Decreases in GlycA were associated with decreases in 2-hour glucose (p<0.008) and BMI (p<0.03).	Obese, prediabetic adolescents	Olson et al. 2018[38]

**To explore the effect of bariatric surgery on GlycA in severely obese adults.**	23 obese non-diabetic women undergoing Roux-en-Y gastric bypass. 31 obese non-diabetic women with sleeve gastrectomy.<br>14 non-obese controls.<br>Baseline, 6- and 12-month analysis	NMR-algorithm at LabCorp, Inc. (formerly LipoScience)[14]<br>Plasma samples	Bariatric surgery significantly reduced GlycA by 6 months (451 ± 47 µmol/L vs. 383 ± 50 µmol/L; P < 0.001) with further reduction at 12 months (348 ± 41 µmol/L; P < 0.001) and no difference between procedures. Increased high density lipoprotein particle size was strongly associated with reduced GlycA.	Bariatric surgery for severe obesity	Manmadhan et al. 2019[39]
**To contrast whether metabolic phenotyping can provide a better understanding of the unique set of regulatory perturbations that predispose to diabetes and its associated complications/ comorbidities.**	38 diabetes patients with good glycaemic control (DB).<br>35 patients with diabetes complications with inadequate glycaemic control (DC).<br>50 healthy controls.	Bruker Biospin Avance-III 800 MHz<br>CPMG pulse<br>Serum samples	Residual signals of N-acetyl glycoproteins (NAG) were found to be decreased in patients with diabetes complications compared to diabetes patients and healthy controls.	Diabetes and diabetes complications	Rawat et al. 2019[40]
**To study the relation between GlycA and type 2 diabetes and compare it with high-sensitivity C-reactive protein.**	26,508 women with a follow-up of 17.2 y., 2,087 T2DM cases (WHS study)	NMR-algorithm at LabCorp, Inc. (formerly LipoScience)[14]<br>Plasma samples	The relative risk of GlycA with type 2 diabetes was somewhat higher for individuals with baseline BMI < vs =25 kg. Both GlycA and hsCRP were significantly associated with the risk of incident type 2 diabetes.	T2DM	Akinkuolie et al. 2015[41]

**To investigate whether intestinal microbiota composition and serum metabolic and inflammatory profiles differ and are interrelated in overweight and obese women during early pregnancy.**	52 overweight and 47 obese pregnant women in early pregnancy	GlycA-Brainshake LTD (now Nightingale Health LTD protocol)[15,17] Serum samples	Low-grade inflammatory markers, GlycA and hsCRP, were statistically significantly higher in obese pregnant women than in overweight pregnant women. The correlation coefficients were also higher between GlycA and lipids than between hsCRP and lipids. GlycA and hsCRP correlated with the following concentrations; isoleucine, leucine and phenylalanine. GlycA also correlated with alanine.	Overweight and obese pregnant women	Houttu et al. 2018[42]
**To investigate the associations of GlycA, interleukin-1 receptor antagonist (IL-1RA), and high-sensitivity C-reactive protein (hs-CRP) with insulin secretion, insulin sensitivity, incident type 2 diabetes, hypertension, CVD events, and total mortality in the prospective METSIM study.**	5401 men without diabetes at baseline or during the follow-up period (6.8 years)	Bruker AVANCE III 500/600 MHz spectrometer Serum samples	During the follow-up period GlycA was associated with impaired insulin secretion, hyperglycemia, incident type 2 diabetes and CVD.	Insulin sensitivity and secretion	Filezova et al. 2017[43]

**To examine the relation of GlycA, GlycB, and CRP with direct measures of insulin sensitivity (insulin sensitivity index [SI]) and insulin secretion (acute insulin response [AIR]).**	1,225 participants (278 with T2DM and 947 without diabetes) in the IRAS.	NMR-algorithm at LabCorp, Inc. (formerly LipoScience)[14] Plasma samples	1) Adiposity and SI have independent relationships with CRP concentration and GlycA and GlycB NMR signals; 2) Both CRP and GlycA demonstrate a statistically independent relation to insulin SI, suggesting that GlycA may reflect an inflammatory pathway distinct from the pathway related to CRP: 3) All three inflammatory markers are more related to 2-h glucose than to fasting glucose. 4) GlycB has weaker relationships with CRP and measures of insulin resistance and adiposity than GlycA.	Insulin resistance and insulin secretion	Lorenzo et al. 2017[44]
**To investigate how obesity, insulin resistance and low-grade inflammation link to circulating metabolites, and whether the connections are due to genetic or environmental factors.**	1368 (531 monozygotic (MZ) and 837 dizygotic (DZ)) twins of healthy young adults. FinnTwin16 and FinnTwin12 cohorts	Bruker AVANCE III spectrometer operating at 500 MHz Serum samples	Fat, especially in the abdominal area, together with HOMA-IR and CRP correlated significantly with an atherogenic lipoprotein profile, higher levels of branched-chain and aromatic amino acids, higher levels of **glycoprotein**, and a more saturated fatty acid profile.	Obesity, insulin resistance , low-grade inflammat ion	Bogl et al. 2016[45]

**To evaluate how exercise-based lifestyle or exercise plus diet interventions modulate GlycA in persons at risk of T2DM.**	169 sedentary adults with prediabetes. 6-month exercise-based lifestyle interventions, 4 intervention groups: (1) low amount/moderate intensity (2) high-amount/moderate intensity (3) high-amount/vigorous-intensity (4) a Clinical Lifestyle (combined diet plus low-amount/moderate-intensity exercise)	NMR-algorithm at LabCorp, Inc. (formerly LipoScience)[14] Plasma samples	At baseline, women had significantly greater concentrations of GlycA. No significant differences between groups were detected. GlycA was reduced on average by 3% in the High-Vig group and on average by 4% in the Clinical Lifestyle intervention. The Low-Mod group reduced GlycA on average by 1% while the High-Mod group increased GlycA on average by 1%.	Prediabetes /exercise and diet-based lifestyle interventions	Barlett et al. 2017[46]
**To evaluate the analytical performance of the GlycA test, measured on the Vantera® Clinical Analyzer. To study the relationship of GlycA with the risk of T2DM.**	4524 individuals from the PREVEND study (general population) Follow-up 8.5 years	Reference: GlycA-LipoScience protocol Comparator: Vantera® Clinical Analyzer, a 400 MHz NMR spectrometer Plasma samples	Participants with higher levels of GlycA were more likely to be older and tended to have higher BMI, blood pressure, glucose and hsCRP levels. GlycA predicted incident T2DM in models adjusted for age, sex, and for BMI, alcohol intake, smoking status, lipid lowering drugs, anti-hypertensive medication, systolic blood pressure, total cholesterol, HDL-C and TG.	T2DM	Connelly et al. 2016[34]

**To compare plasma GlycA and Lp-PLA2 mass between subjects without T2DM or MetS and subjects with T2DM and/or MetS**	58 subjects with T2DM and/or MetS (group 2)<br>40 control subjects (group 1)	NMR-algorithm at LabCorp, Inc. (formerly LipoScience)[14]<br>Plasma samples	GlycA and hsCRP were higher, whereas Lp-PLA2 was lower in group 2 vs group 1. GlycA was positively related to hsCRP in each group.	T2DM and/or MetS	Gruppen et al. 2016[47]
**To investigate associations of circulating metabolites from high-throughput profiling separately for fasting and 2-h glucose cross-sectionally and prospectively in middle-aged Finnish men and women**	1873 individuals<br>618 after 6.5 years	Bruker AVANCE III spectrometer operating at 500.36 MHz<br>Serum samples stored at -80ºC (NMR Spectroscopy protocol by Soininen et al. 2009)[15]	A1-acid glycoprotein was prospectively associated with both fasting and 2h-glucose ($p<0.05$)	Glycemia	Würt et al. 2012[48]
**(i) To determine whether plasma GlycA is elevated in subjects with MetS and (ii) to assess the relationship of GlycA with plasma LCAT activity**	58 MetS (46 subjects with T2DM) and 45 controls	NMR-algorithm at LabCorp, Inc. (formerly LipoScience)[14]<br>Plasma samples	GlycA was found to be elevated in MetS, but not positively associated with the presence of T2DM. GlycA was correlated positively with systolic blood pressure, BMI and waist circumference as well as with plasma triglycerides, and inversely with HDL cholesterol. GlycA was related to higher plasma LCAT activity.	MetS	Gruppen et al. 2015[49]

**To compare GlycA and other markers of inflammation among hospitalized, noncritically ill patients with type 2 diabetes**	121 T2DM patients: (71 CHF, 21 cardiac-non-CHF, 18 infectious diseases, and 11 in other categories)	NMR-algorithm at LabCorp, Inc. (formerly LipoScience)[14] Plasma samples	GlycA varied significantly across diagnostic categories and values were highest in patients with infectious disease. GlycA was associated with higher IL-6 and CRP and lower hemoglobin and estimated GFR; GlycA was not associated with HbA1c.	T2DM and other noncritica l illnesses	Dungan et al. 2015[50]
**To test whether plasma GlycA elevations are associated with lower bilirubin in MetS, and to assess the extent to which the association of GlycA with MetS is attenuated when taking account bilirubin levels.**	58 MetS (46 T2DM) 63 without MetS (19 T2DM)	NMR-algorithm at LabCorp, Inc. (formerly LipoScience)[14] Plasma samples	GlycA and hs-CRP were higher, coinciding with lower bilirubin in MetS ($p<0.01$ for each). GlycA was strongly correlatedwith hs-CRP. GlycA and hs-CRP were both associated positively with the presence of MetS. GlycA and hs-CRP were negatively related to bilirubin, regardless of MetS and diabetes status.	MetS	Dullaart et al. 2015[51]

1S.3. Cardiovascular risk					
**Main objective**	Participants	Key methods	Main results related to glycoproteins	Clinical topic	Reference
**A new binning algorithm, Adaptive Intelligent Binning (AI-Binning), is presented to characterize hypertensive spectra**	40 hypertensive and 40 matched normotensive subjects	Bruker Avance II spectrometer 700.13 MHz CMPG pulse Serum samples	The binning algorithm enabled the relevant metabolites to be identified and suggested the involvement of a-1 acid glycoproteins and choline biochemistry in hypertension.	HBP	Tim De Meyer - 2008[20]

**To evaluate changes in key metabolites following congenital heart surgery and to examine the potential of metabolic profiling for stratifying patients in terms of expected clinical outcomes.**	28 children undergoing surgery for congenital heart disease (15 underwent tight glycemic control postoperatively and 13 were treated conventionally)	Bruker AVANCE III spectrometer 600 MHz CPMG pulse Plasma samples	Acetate, acetoacetate, acetone, alanine, citrate, formate, glucose, 3-hydroxybutyrate, isoleucine, leucine, N-acetylated glycoprotein, threonine, and valine had significantly higher concentrations in the postoperative samples.	Children with congenital heart disease	Correia et al. 2015[21]
**To investigate the associations of plasma metabolic markers with the risk of incident MI, IS and ICH.**	912 MI, 1146 IS, and 1138 ICH cases 1466 control subjects	GlycA-Brainshake LTD (now Nightingale Health LTD protocol)[15,17] Serum samples	Glycoprotein acetyls, ketone bodies, glucose, and docosahexaenoic acid were associated with all 3 diseases	CVD prediction	Holmes et al. 2018[52]
**To determine if GlycA adds independent value to hsCRP for CV risk prediction.**	2996 patients in the Intermountain Heart Collaborative Study who underwent coronary	NMR-algorithm at LabCorp, Inc. (formerly	GlycA and HS-CRP were moderately correlated. The interaction between GlycA and HS-CRP was statistically significant for the outcome of death. Baseline levels of both GlycA and HS-CRP were found to be	CVD prediction	Muhlestein et al. 2018[53]

	angiography. Median follow-up 7.9 years	LipoScience)[14] Plasma samples	independent and additive markers of risk for future major adverse CV events, especially death and hospitalization.		
**a) To define the role of GlycA as a potential biomarker of adverse events in patients undergoing cardiac catheterization; (b) to evaluate the independent and incremental predictive performance of GlycA and HDL subclasses; and (c) to understand a priori defined potential interactions between HDL subclasses and GlycA.**	7617 individuals in the CATHGEN cardiac catheterization biorepository	NMR-algorithm at LabCorp, Inc. (formerly LipoScience)[14] Plasma samples	GlycA was associated with the presence and extent of coronary artery disease and with all-cause mortality, cardiovascular mortality and noncardiovascular mortality in models adjusted for 10 cardiovascular risk factors. GlycA and smaller HDL subclasses had independent but opposite effects on mortality risk prediction, with smaller HDL subclasses being protective. Individuals without diabetes who had the greatest quartile of GlycA concentration actually had a greater risk than patients with diabetes who had a lower concentration of GlycA	CVD prediction	McGarrah et al. 2017[54]

**To evaluate the association and interaction between various HDL sub-particles, the inflammatory marker GlycA, and future cardiovascular risk.**	2,848 patients from the angiography registry of the Intermountain Heart Study	NMR-algorithm at LabCorp, Inc. (formerly LipoScience)[14] Plasma samples	GlycA, small HDL-P and medium HDL-P were significantly associated with cardiac death, but large HDL-P was not after adjustment for CV risk factors and medications. Only small HDL-P had a significant interaction with GlycA.	CVD prediction	Muhlestein et al. 2016[55]
**To test if GlycA is associated with incident CVE and can improve CV risk prediction with traditional risk factors.**	6,939 individuals	NMR-algorithm at LabCorp, Inc. (formerly LipoScience)[14] Plasma samples	GlycA was strongly associated with incident CVE after adjustment for clinical risk factors. Consideration of GlycA in addition to traditional risk factors improves CV risk prediction in a high-risk population.	CVE	McGarrah et al. 2015[56]
**To study the association of GlycA and GlycB with CVD.**	2,996 patients who underwent coronary angiography for CAD determination/follow-up	NMR-algorithm at LabCorp, Inc. (formerly LipoScience)[14] Plasma samples	No significant association was found between GlycA or GlycB and CAD. The incidence of major adverse CV events was significantly higher in patients with higher levels of GlycA and GlycB.	CVD prediction	Mulhestein et al. 2014[57]

**To examine the association of baseline GlycA concentration with incident CVD events. To assess whether GlycA provided additional clinical utility for the risk of future cardiovascular events beyond the information conveyed by hsCRP**	27,491 initially healthy women. Follow-up 17.2 years for CVD events (JUPITER trial)	NMR-algorithm at LabCorp, Inc. (formerly LipoScience)[14] Plasma samples	At baseline, increasing quartiles of GlycA were associated with a higher prevalence of traditional CVD risk factors and higher concentrations of hsCRP. GlycA and hsCRP were moderately correlated. The association of GlycA with CVD was attenuated after adjusting for hsCRP	CVD	Akinkuolie et al. 2014[58]
**To identify biomarkers for all-cause mortality and enhance risk prediction. A high-throughput profiling of 106 plasma sample biomarkers are quantified by NMR**	17,345 individuals	(NMR spectroscopy protocol by Soininen et al. 2009)[15] Plasma and serum samples	Alpha-1-acid glycoprotein, albumin, VLDL size, and citrate biomarkers were predictive of cardiovascular mortality, death from cancer and other nonvascular diseases. Alpha-1-acid glycoprotein was the strongest multivariate predictor of the risk of death from all causes	All-cause mortality	Fischer et al. 2014[59]
**To determine differences in life expectancy in men and women from the PREVEND cohort with higher vs. lower levels of GlycA and hsCRP**	5526 subjects from PREVEND study Median *follow up* 8.5 years	Vantera® Clinical Analyzer, a 400 MHz NMR spectrometer Plasma samples	Life expectancy in men and women at the end of follow up was lower in the highest vs the lower three quartiles of GlycA ($P < .001$). For hsCRP, this was only observed in men ($P < .001$) but not in women ($P=0.67$).	Life expectancy	Gruppen et al.2019[60]
**To examine the effects of ERN treatment on lipoprotein particles and GlycA and their relations with incident CVD events including mortality in**	3,414 AIM-HIGH participants	NMR-algorithm at LabCorp, Inc. (formerly LipoScience)[14] Plasma samples	Compared to placebo, ERN treatment lowered VLDL and LDL and increased HDL particle concentrations, increased LDL and HDL particle sizes, but did not affect GlycA. Baseline and in-trial GlycA levels were associated with increased risk of CVD	CVD prediction	Otvos et al. 2018[61]

**a substudy analysis of the AIM-HIGH trial**			events. None of the lipoprotein particle classes or subclasses were associated with incident CVD. All-cause mortality was significantly associated with both GlycA and low levels of small HDL particles.		
**To determine whether GlycA levels were associated with CV health as defined by the LS7 score and with each of its individual seven health metrics**	6,479 MESA participants without CVD	NMR-algorithm at LabCorp, Inc. (formerly LipoScience)[14] Plasma samples	After multivariable adjustment, GlycA remained independently and inversely associated with CV health categories. For each of the individual LS7 metrics (Blood Pressure, Blood Glucose, Total Cholesterol, Smoking, Diet, Physical Activity, and BMI), there was an inverse significant relation with GlycA levels.	CV Health	Benson et al. 2018[62]
**To decompose the spectral GlycA biomarker by developing imputation models for GlycA's constituent glycoproteins, and use these imputed molecular phenotypes to investigate associations with disease risk.**	11,861 adults across two population-based cohorts (DILGOM07 and FINRISK) Median 8 years follow-up	GlycA-Brainshake LTD (now Nightingale Health LTD protocol)[15,17] + immunoassays for AAT, AGP, HP, and TF + imputation models (Machine learning) Serum samples	Imputed AAT was significantly associated with risk of hospitalisation or death for substantially more outcomes (including liver diseases, heart failure and COPD). In contrast, imputed AGP was significantly associated with increased risk from only two outcomes: heart failure and chronic lower respiratory diseases. HP was the strongest predictor of chronic lower respiratory diseases, inflammatory polyarthropathies and atherosclerosis. Multiple individual glycoproteins independently and weakly predict each disease, with the GlycA NMR signal capturing this risk in aggregate.	Morbidity and mortality	Ritchie et al. 2018[63]

**To examine the longitudinal association between GlycA and mortality among initially-healthy individuals.**	27,524 participants in the WHS follow-up Replicate in 12,527 individuals JUPITER trial	NMR-algorithm at LabCorp, Inc. (formerly LipoScience)[14] Plasma samples	GlycA for all-cause mortality was significantly increased at 5 years. Similar risk for all-cause mortality was observed in the replication cohort. Risk of CVD and cancer mortality was increased at 5 years	All-cause mortality	Lawler et al. 2016[64]

1S.4. HIV infection

**Main objective**	Participants	Key methods	Main results related to glycoproteins	Clinical topic	Reference
**To examine the associations between GlycA and subclinical coronary plaque among HIV-infected and HIV-uninfected men participating in MACS.**	935 men from MACS (63% HIV-infected individuals)	NMR-algorithm at LabCorp, Inc. (formerly LipoScience)[14] Plasma samples	1) Higher quartiles of plasma GlycA were significantly positively associated with HCV infection, HIV infection, levels of hsCRP, IL-6, fibrinogen, sCD163, sCD14, CCL2 and CAC.<br>2) GlycA levels were higher in HIV-infected men than in HIV-uninfected men (397±68 vs 380±60 μmol/L, p=0.0001), and higher for men with detectable vs. undetectable viral load (413±79 vs 393±65 μmol/L, p=0.004).<br>3) Plasma GlycA levels positively correlated with smoking pack-years and	CVD in HIV-infection	Tibuakuu et al. 2018[65]

			HCV infection status and inversely correlated with HDL cholesterol levels and physical activity level among HIV-infected participants.<br>4) After adjusting for HIV serostatus, demographic and CVD risk factors, GlycA level was associated with a higher prevalence of CAC and coronary stenosis.<br>5) Among men with plaque, GlycA was positively associated with the extent of CAC and total plaque.		

1S.5. Chronic inflammatory diseases					
**Main objective**	Participants	Key methods	Main results related to glycoproteins	Clinical topic	Reference
**To determine whether an H-NMR spectroscopic metabolic phenotypin approach could be used to identify signatures reflective of the dynamic, pathological metabolic perturbations associated with fibrosis in CHC patients**	50 CHC patients<br>63 CHC patients validation	Bruker Avance (Avance III) 600 MHz<br>NMR spectrometer<br>CPMG pulse<br>Plasma samples	Increased severity of fibrosis was associated with higher tyrosine, phenylalanine, methionine, citrate and, very-low-density lipoprotein (vLDL) and lower creatine, low-density lipoprotein (LDL), phosphatidylcholine, and NAC	CHC	Sands et al. 2015[28]

**To investigate GlycA levels in a cohort of healthy individuals, patients with CD and patients with UC prior to and after therapeutic control of inflammation.**	37 Crohn's disease patients and 21 ulcerative colitis patients before and after biologic therapy (ADM, IFX, VDM, UST)<br>10 healthy controls.	GlycA-Brainshake LTD (now Nightingale Health LTD protocol)[15,17]<br>Serum samples	GlycA levels were significantly higher in patients with active IBD (CD and UC) than in healthy controls. GlycA levels from CD and UC patients dropped to control levels after mucosal healing. Only GlycA post-treatment levels consistently showed a significant difference between responder and non-responder levels	IBD (CD and UC)	Dierckx et al. 2018[66]
**To characterize the plasma glycoprotein profile of a cohort of patients with RA versus healthy individuals and to model the activity of RA to identify patterns indicating the severity of the disease**	210 RA patients<br>203 healthy individuals	Bruker Avance III<br>600 spectrometer<br>NOESY, LED<br>Plasma samples	RA patients presented significantly higher GlycA area and H/W GlycA and GlycB ratios than the control population. GlycA and GlycB variables derived from 1H NMR, along with classic inflammatory parameters, help to improve the classification of individuals with high RA disease activity based on DAS28.	RA	Fuertes-Martín et al. 2018[67]
**To investigate if GlycA could be<br>associated with lupus nephritis severity**	105 active SLE patients<br>39 quiescent SLE patients<br>21 non-lupus nephritis controls<br>29 healthy controls	GlycA-Brainshake LTD (now Nightingale Health LTD protocol)[15,17]<br>Serum samples	GlycA was correlated to C-reactive protein (CRP), neutrophil count, proteinuria and the SLE disease activity index.<br>Patients with active SLE showed significantly higher GlycA concentration than healthy controls (p=0.009), non-lupus nephritic controls (p=0.04) and quiescent SLE patients (p<10-6). In patients with biopsy-proven active LN, GlycA was higher in proliferative than non-proliferative lupus nephitis	SLE and Lupus nephritis	Dierckx et al. 2018[68]

**To conduct a plasma metabolomic analysis to determine the characteristics of patients with COPD with abnormal Savda syndrome using NMR spectroscopy technology**	103 COPD patients mild (n=15), moderate (n=38) and severe (n=50)	Inova 600, Varian Medical Systems Spectrometer CPMG pulse Plasma samples	The concentration of metabolites such as glycoprotein in the plasma of patients with COPD with abnormal Savda syndrome was lower than in the plasma of patients with COPD with non-abnormal Savda syndrome and the plasma of healthy subjects	Savda syndrome in COPD	Xu et al. 2015[69]
**To investigate the association of GlycA with albuminuria and eGFR in a Brazilian cohort of middle-aged men and women.**	5050 participants from ELSA-Brasil Study	NMR-algorithm at LabCorp, Inc. (formerly LipoScience)[14] Plasma samples	GlycA was higher in older women, smokers, teetotallers, the obese and those with diabetes, hypertension or dyslipidemia. GlycA was independently associated with log albuminuria and inversely related to eGFR.In the ROC curve, GlycA had a higher AUC than hsCRP (AUC 0.67 vs. 0.62, p = 0.06) for the association with albuminuria A2 or A3.	CKD	Titan et al. 2017[70]
**To examine whether GlycA levels increased with active disease and to establish whether this could be a more useful biomarker for predicting cardiovascular events in lupus.**	52 patients 229 follow-up visits	NMR-algorithm at LabCorp, Inc. (formerly LipoScience)[14] Plasma samples	Mean GlycA levels in this cohort were higher than those reported for a normal population. GlycA increased significantly with each point increase in SLEDAI- The African American population had lower VLDL, triglycerides and higher levels of GlycA than the other SLE groups.	SLE	Durcan et al. 2016[71]

**To investigate the relationships between GlycA and psoriasis, and between GlycA and subclinical CVD**	122 psoriasis patients<br>109 controls	NMR-algorithm at LabCorp, Inc. (formerly LipoScience)[14]<br>Plasma samples	Psoriasis patients had higher levels of hsCRP and GlycA which remained significant after adjustment for age, sex, BMI and traditional CV risk factors. GlycA is associated with vascular inflammation and coronary artery disease. Anti-TNF therapy decreases GlycA levels.	Subclinical CVD in psoriasis	Joshi et al.2016[72]
**To explore the relationships of GlycA with inflammation and cardiometabolic risk in RA, and explore whether these relationships were similar to those of people without RA.**	50 mild-moderate RA patients<br>39 controls	NMR-algorithm at LabCorp, Inc. (formerly LipoScience)[14]<br>Plasma samples	GlycA concentrations were significantly elevated in RA versus controls (P = 0.036). In RA, greater GlycA associated with disease activity and inflammation. In BMI-matched controls, these inflammatory associations were absent or weaker. In RA, greater GlycA was associated with more total abdominal adiposity and less muscle density. In BMI-matched controls, GlycA was associated with more cardio-metabolic markers: BMI, waist circumference, adiposity measures and insulin resistance	RA and CVD	Barlett et al. 2016[73]
**To test whether GlycA is a biomarker of disease activity and is associated with coronary artery atherosclerosis in patients with RA.**	166 RA patients<br>90 control subjects	NMR-algorithm at LabCorp, Inc. (formerly LipoScience)[14]<br>Plasma samples	GlycA concentrations were higher in patients with RA than in control subjects. In RA, GlycA was strongly correlated with DAS28 based on erythrocyte sedimentation rate (DAS28-ESR) and DAS28 based on C-reactive protein (DAS28-CRP) and their components, including tender and swollen joint counts, global health score, ESR and CRP. For each quartile increase in GlycA, the odds of having coronary artery calcium increased by 48 %.	RA	Ormseth et al. 2015[74]

**To test the hypothesis that GlycA concentrations are elevated in patients with SLE and associated with other markers of inflammation and coronary atherosclerosis.**	116 SLE patients<br>84 control subjects	NMR-algorithm at<br>LabCorp, Inc. (formerly LipoScience)[14]<br>Plasma samples	Patients with SLE had higher concentrations of GlycA than control subjects. In patients with SLE, concentrations of GlycA were significantly associated with ESR, CRP, e-selectin, intracellular adhesion molecule-1, and triglycerides.	SLE	Chung et al.2016[75]
**To characterize biological processes associated with GlycA by leveraging population-based omics data and health records from >10,000 individuals.**	11,825 individuals from 3 cohorts: DILGOM07 (300 female), +FINRISK (7599 individuals) and + YFS (3596 individuals follow-up)	Bruker AVANCE III, 500 MHz spectrometer (the 3 cohorts)<br>Serum samples	In apparently healthy individuals, GlycA can be chronically elevated for periods of up to a decade. In individuals with high GlycA, modest elevation of numerous cytokines is suggestive of a prolonged low-grade inflammatory state.<br>High GlycA levels correlated with an increased risk of hospitalization and death from various infections (septicemia and pneumonia). GlycA levels persists for over a decade.	Chronic inflammation and long-term risks	Ritchie et al. 2015[76]
**1H NMR spectroscopy-based metabolic phenotyping was used to identify biomarkers in the plasma of patients with RA.**	47 RA patients<br>51 healthy subjects	Bruker Avance 600 MHz spectrometer<br>Plasma samples stored at or below -25 °C for a period of up to 19 months	Cholesterol, lactate, acetylated glycoprotein, and lipid signatures were found to be candidate biomarkers for disease severity.	RA	Lauridsen et al. 2010[5]

1S.6. Cognitive function and psychological health					
**Main objective**	Participants	Key methods	Main results related to glycoproteins	Clinical topic	Reference
**To present a new three-molecular-window approach that gives specific molecular data on macromolecular lipid–protein aggregates such as lipoprotein particles, on various low-molecular-weight metabolites, and on individual lipid molecules together with their degree of (poly)(un)saturation.**	180 elderly people (54 related to MCI, with severely increased risk of AD).	AVANCE 500 DRX spectrometer<br>1D CPMG pulse sequence<br>Serum samples	Positive association between MCI and the MetS. Low relative amount of n3 fatty acids appears more indicative of MCI than low serum n3 or polyunsaturated fatty acid concentration as such. Elevated circulating glycoproteins in the risk of AD.	MCI and AD	Tukiainen et al 2008[77]
**To develop a biomarker panel to provide support for objective diagnostic laboratory tests for psychological suboptimal health.**	22 psychological suboptimal health patients<br>23 healthy controls	Bruker 600 MHz AVANCE III NMR spectrometer<br>CPMG<br>Plasma samples	A biomarker panel containing phenylalanine, glutamine, tyrosine, citrate, N-acetyl-glycoproteins and TMAO was identified and there was a high correlation with the state of psychological suboptimal health.	Psychological suboptimal health	Tian et al.2016[29]
**To examine the association of the inflammatory markers CRP, fibrinogen, WBC and GlycA, measured in young**	507 participants (13 years follow up)	NMR-algorithm at LabCorp, Inc. (formerly	The highest quintile of GlycA change, but not the baseline inflammation measures, was inversely related to global cognition as	Cognitive function	Cohen-Manheim et al. 2015[78]

**adulthood and of GlycA change over 13 years follow-up with cognitive function in midlife**		LipoScience)[14] Plasma samples	well as to information processing speed and memory domains.		
1S.7. Rare vascular diseases					
**Main objective**	Participants	Key methods	Main results related to glycoproteins	Clinical topic	Reference
**To confirm previous TA findings in a larger group of patients and to study their correlation with disease activity**	Total: 45 active TA patients and 53 inactive TA patents 57 TA patients (active and inactive) follow-up 3.1 months 43 healthy controls	Bruker Avance III 800 MHz NMR spectrometer CPMG pulse Serum samples	The sera of TA patients were characterized by elevated levels of LDL, NAG, glucose, glutamate, phosphoglyceride, glycerol, glycerophophocholine, and decreased levels of glucogenic amino acids, lactate and creatine. The key metabolites with highest discriminatory potential in active TA were glutamate and N-acetyl glycoprotein (NAG), both elevated.	TA	Jain et al. 2018[30]
**To determine the ability of GlycA concentrations and NMR lipoprotein particle measures to distinguish pediatric patients with acute KD from those with other febrile illnesses.**	75 acute KD 36 post-treatment subacute KD 63 convalescent KD 48 febrile controls 48 and age-similar healthy controls	NMR-algorithm at LabCorp, Inc. (formerly LipoScience)[14] Plasma samples	GlycA was higher in acute KD subjects than other groups. GlycA and NMR-measured lipoprotein particle parameters may be useful for distinguishing acute KD from bacterial or viral illnesses (ROC AUCs of 0.910 and 0.909 for GlycA combined with either the LDL-P/HDL-P ratio or LDL-P, respectively).	Kawasaki disease	Connelly et al. 2016[79]
**To investigate the metabolic profiles of sera derived from TA patients using NMR with**	29 TA patients 30 controls	Bruker Avance III 800 MHz NMR	Compared to healthy controls, TA patients had (a) increased serum levels of choline metabolites, LDL cholesterol, NAGs, and	TA	Guleria et al. 2015[80]

**an aim to assess (a) whether NMR-based serum metabolomics would allow early identification of TA patients and (b) whether metabolic differences in TA patients are related to the risk of TA progression.**		spectrometer CPMG pulse Serum samples	glucose and (b) decreased serum levels of lactate, lipids, HDL cholesterol, and glucogenic amino acids.		

1S.8. Pregnancy

**Main objective**	Participants	Key methods	Main results related to glycoproteins	Clinical topic	Reference
**To explore the effects of habitual diet and adherence to the recommended diet on gut microbiota, serum lipidomics and low-grade inflammation, and the relationship of gut microbiota composition to serum lipidomics and inflammatory markers in overweight and obese pregnant women.**	100 overweight women in early pregnancy	GlycA-Brainshake LTD (now Nightingale Health LTD protocol) [15,17] Serum samples	Multiple nutrients correlated with GlycA including fibre, LC-PUFA and n-3 LC-PUFA and several vitamins and minerals but no correlations were detected between any of the nutrients and hs-CRP and LPS. Higher gut microbiota richness is negatively linked with low-grade inflammation marker GlycA, which was further related to intake of several nutrients including fibre and LC-PUFA. No similar relationship between hs-CRP and gut microbiota richness, suggesting that the inflammatory pathway of GlycA is different from that of CRP.	Diet in overweight pregnant women	Röytiö et al. 2017[81]

**To investigate the extent to which intestinal permeability, measured by serum zonulin concentration, is related to metabolic endotoxemia and metabolic risk markers in overweight pregnant women.**	100 overweight women in early pregnancy	GlycA-Brainshake LTD (now Nightingale Health LTD protocol) [15,17] Serum samples	Both LPS and GlycA showed a positive relationship with insulin resistance, serum insulin, triglycerides, and total and LDL-cholesterol, and a negative relationship with insulin sensitivity. Serum zonulin was found to associate positively with LPS, hs-CRP, GlycA, insulin, HOMA2-IR, triglycerides, and total cholesterol.	Intestinal permeabil ity in overweig ht pregnant women	Mokkala et al. 2017[82]
**To evaluate the metabolic adaptations reflected in plasma throughout healthy pregnancies by carrying out an untargeted 1H NMR study**	20 non-pregnant women<br>25 1st T<br>30 2nd T<br>12 3rd T<br>7 post-delivery	Bruker Avance DRX 500 spectrometer Plasma samples Noesy, cpmg and led pulse sequences	Gradually increase of N-acetyl glicoproteins.<br>Direct link between LDL+VLDL and N-acetyl-glycoproteins	Pregnanc y	Pinto et al. 2015[33]

1S.9. Primary aldosteronism					
**Main objective**	Participants	Key methods	Main results related to glycoproteins	Clinical topic	Reference
**To determine the extent to which (apo)lipoproteins, lipoprotein particle concentrations, GlycA and BCAA, as determined by NMR spectroscopy, were altered in individuals with PA, compared to non-hypertensive control subjects,**	20 primary aldosteronism patients 2,819* control subjects without hypertension<br>501* subjects with untreated hypertension	Vantera® Clinical Analyzer, a 400 MHz NMR spectrometer Plasma samples	GlycA was increased in PA vs the three groups ($P < 0.016$).	Primary aldostero nism	Berends et al. 2019[83]

**subjects with untreated hypertension and subjects with medically treated hypertension.**	878* subjects with treated hypertension *From the PREVEND study				

1S.10. Sickle cell disease					
**Main objective**	Participants	Key methods	Main results related to glycoproteins	Clinical topic	Reference
**To evaluate plasma GlycA levels in a cross-sectional sample of patients with SCD and specifically test levels in patients experiencing an acute painful vaso-occlusive crisis.**	488 patient with SCD in "steady state" including 52 healthy controls and 12 patients (from the same group) during an acute pain crisis.	Vantera® Clinical Analyzer, a 400 MHz NMR spectrometer Plasma samples	The mean plasma GlycA level was lower in SCD than in healthy controls. Within the same patient, mean plasma GlycA during acute pain crisis was lower than in steady state, although the difference was not significant.	SCD	Weisman et al. 2018[84]

1S.11. Human African Trypanosomiasis					
**Main objective**	Participants	Key methods	Main results related to glycoproteins	Clinical topic	Reference
**To characterize the metabolic effects of T. brucei rhodesiense infection in humans**	46 HAT patients 21 controls	Bruker Avance 600 MHz CPMG Plasma samples	Among other metabolites, NAG is significantly higher in disease ($p<0.01$)	HAT	Lamour et al. 2015[85]

1S.12. Sodium intake					
**Main objective**	Participants	Key methods	Main results related to glycoproteins	Clinical topic	Reference
**To investigate the association of 24-h sodium excretion with the 2 inflammatory markers GlycA and hsCRP in a large population-based cohort of men and women. To assess the role of adiposity in the association between sodium intake and inflammatory markers**	3,935 subjects from a general population (PREVEND study)	NMR-algorithm at LabCorp, Inc. (formerly LipoScience)[14] Plasma samples	The proinflammatory biomarkers GlycA and hsCRP are inversely related to higher 24-h sodium excretion when taking into account the variation in adiposity.	Sodium intake	Gruppen et al. 2016[86]

1S.13. Tobacco smoking					
**Main objective**	Participants	Key methods	Main results related to glycoproteins	Clinical topic	Reference
**To determine whether smoking is associated with systemic inflammation as measured by GlycA levels. We also sought to compare the strength of the association of smoking and GlycA versus the association of smoking and hsCRP.**	11,509 participants (6,774 from the MESA and 4,735 from ELSA-Brasil)	NMR-algorithm at LabCorp, Inc. (formerly LipoScience)[14] Plasma samples	Compared with people who had never smoked, former and current smokers had significantly higher adjusted means of GlycA levels. Each 5-unit increase in pack-years of smoking was associated with higher GlycA levels among former and current smokers. Among former smokers, each 5-year increase in time since quitting smoking was associated with lower GlycA levels and each 10-unit increase in number of cigarettes/days was associated with higher GlycA among current smokers.	Smoke	Kianoush et al. 2017[87]

			Results were significant for the association between all measures of smoking behaviour and GlycA and hsCRP.		

1S.14. Effect of excercise					
**Main objective**	Participants	Key methods	Main results related to glycoproteins	Clinical topic	Reference
**To study whether persistent physical activity compared with inactivity has a global effect on serum metabolome and leads to reduced cardiometabolic disease risk**	16 same-sex twin pairs >30-year discordance for physical activity and 1,037 age-and sex-matched pairs. Median follow-up 5 years	Bruker AVANCE III 500 MHz spectrometer NOESY and CPMG pulses Serum samples	Isoleucine, α1-acid glycoprotein, and glucose were lower in the physically active than in the inactive individuals (P<0.001 in meta-analysis) (findings persisted after adjustment for BMI).	Exercise interventi ons	Kujala et al. 2013[18]
**To examine the effects of regular exercise on the inflammatory marker GlycA across seven studies and 14 exercise interventions**	1,568 individuals	NMR-algorithm at LabCorp, Inc. (formerly LipoScience)[14] Plasma samples	Regular exercise significantly reduced plasma GlycA even after adjustment for age, sex, race, baseline BMI, and baseline GlycA. Changes in GlycA were correlated with changes in traditional inflammatory markers, C-reactive protein, interleukin-6, and fibrinogen. However, these correlations were relatively weak (range r: 0.21e0.38, p < 0.0001).	Exercise interventi ons	Barber et al. 2018[88]

1S.15. Effect of treatments

Main objective	Participants	Key methods	Main results related to glycoproteins	Clinical topic	Reference
**To use 1H NMR and UPLC/MS to study type 2-DM in patients non-treated and treated with metformin hydrochloride**	20 non treated type 2-DM patients<br><br>15 treated type 2-DM	Bruker AV 600 spectrometer CPMG pulse Serum samples	NAC was lower in serum from metformin treated patients than in serum from untreated patients	T2DM treatment	Huo et al 2009[3]
**To understand how markers of inflammation and immune activation change in response to successful ART**	328 HIV-1 infected (week 24 to week 92 follow-up)	NMR-algorithm at LabCorp, Inc. (formerly LipoScience)[14] Plasma samples	On average, a 10% decline but not significant in levels of GlycA was apparent over 48 weeks with all the studied treatment combinations.	ART for HIV-1	Kelesidis et al. 2015[89]

1S.16. Toxicity

Main objective	Participants	Key methods	Main results related to glycoproteins	Clinical topic	Reference
**To evaluate the hepatotoxicity of valproate sodium (antiepileptic drug) through new 1H-NMR markers**	34 epileptic patients	Bruker AV 600 CPMG (64 scans) Serum samples	N-acetyl moieties of glycoprotein significantly increased ($p<0.01$) in valproate sodium induced hepatotoxicity, among other metabolites such as glucose, lactate, acetoacetate, VLDL/LDL, lysophosphatidylcholines, phosphatidylcholines, choline, creatine, amino acids, pyruvate and uric acid.	Drug toxicity	Huo et al. 2014[26]

1S.17. Others					
**Main objective**	Participants	Key methods	Main results related to glycoproteins	Clinical topic	Reference
**To determine potential relationships between GlycA and adiposity, insulin resistance, hs-CRP, leptin, adiponectin, and the leptin/adiponectin ratio, and to test whether GlycA is elevated in subjects with impaired fasting glucose and T2DM.**	103 fasting subjects (30 with normal fasting glucose, 25 with IFG and 48 with T2DM).	NMR-algorithm at LabCorp, Inc. (formerly LipoScience)[14] Plasma samples	Plasma GlycA was correlated positively with BMI, HOMAir, hs-CRP, leptin and the leptin/adiponectin ratio, and inversely with adiponectin. GlycA did not significantly vary with the glucose tolerance category. GlycA was related positively to the leptin/adiponectin ratio, regardless of BMI and HOMAir.	Potential relationships between GlycA and other biomarkers	Dullaart et al. 2015[90]
**a) To report on levels of GlycA and the change in GlycA as children move from 6th to 8th grade; b) to examine whether BMI group is associated with GlycA in these children; c) to determine if fitness was associated with GlycA, LP-IR and traditional lipid panel variables; and d) to examine whether fitness and BMI are independently related to GlycA and/or LP-IR.**	1,664 US adolescents from the HEALTHY study	NMR-algorithm at LabCorp, Inc. (formerly LipoScience)[14] Plasma samples	In the 8th grade the median GlycA values were 27 μmol/L higher for girls than boys. GlycA levels are 19% higher in obese girls than healthy weight girls. Strong evidence ($p<0.001$) that in all sub-groups (6th grade boys and girls, and 8th grade boys and girls) GlycA is higher in higher BMI groups. The lowest levels of GlycA are in the low BMI/high fitness group with the highest levels in the high BMI/low fitness group.	Systemic inflammation in adolescents	Jago et al. 2016[91]

**To use 1H NMR to examine the metabolic profiles of plasma from FD patients before and after treatment by acupuncture.**	6 female FD patients<br>6 female healthy subjects	Varian INOVA 600MHz NMR spectrometer<br>CPMG/BBP-LED<br>Plasma samples	Lower levels of lactate, leucine/isoleucine, NAC, and LDL/VLDL in FD patients than in healthy controls	FD	Wu et al. 2010[6]

Abbreviations: OC, Ovarian cancer; CSCC, Cervical squamous cell carcinoma; BC, Breast cancer; LC, lung cancer; CRC, colorectal cancer; GlcNAc, N-Acetyl glucosamine; TG, total triglycerides; CRP, C-reactive protein; IL-6, interleukin-6; T2DM, type 2 diabetes mellitus; MetS, metabolic syndrome; CVD, cardiovascular disease; CVE, cardiovascular event; COPD, chronic obstructive pulmonary disease; RA, rheumatoid arthritis; SLE, systemic lupus erythematosus; SELDAI, Systemic Lupus Erythematosus Disease Activity Index; IBD, Inflammatory bowel disease; CKD, chronic kidney disease; CHC, chronic hepatitis C; AD, Alzheimer disease; MCI, mild cognitive impairment; TA, Takayasu arteritis; KD, Kawasaki disease; PUFA, polyunsaturated fatty acids; PA, primary aldosterism; SCL, sickle cell disease.

References

1. Kriat, M.; Vion-Dury, J.; Fayre, R.; Maraninchi, D.; Harlé, J.R.; Confort-Gouny, S.; Sciaky, M.; Fontanarava, E.; Viout, P.; Cozzone, P.J. Variations of plasma sialic acid and N-acetylglucosamine levels in cancer, inflammatory diseases and bone marrow transplantation: a proton NMR spectroscopy study. *Biochimie* **1991**, *73*, 99–104.

2. Torri, G.M.; Torri, J.; Gulian, J.-M.; Vion-Dury, J.; Viout, P.; J. Cozzone, P. Magnetic resonance spectroscopy of serum and acute-phase proteins revisited: a multiparametric statistical analysis of metabolite variations in inflammatory, infectious and miscellaneous diseases. *Clin. Chim. Acta* **1999**, *279*, 77–96.

3. Huo, T.; Cai, S.; Lu, X.; Sha, Y.; Yu, M.; Li, F. Metabonomic study of biochemical changes in the serum of type 2 diabetes mellitus patients after the treatment of metformin hydrochloride. *J. Pharm. Biomed. Anal.* **2009**, *49*, 976–982.

4. Kolwijck, E.; Engelke, U.F.; van der Graaf, M.; Heerschap, A.; Blom, H.J.; Hadfoune, M.; Buurman, W.A.; Massuger, L.F.; Wevers, R.A. *N* -acetyl resonances in *in vivo* and *in vitro* NMR spectroscopy of cystic ovarian tumors. *NMR Biomed.* **2009**, n/a-n/a.

5. Lauridsen, M.B.; Bliddal, H.; Christensen, R.; Danneskiold-Samsøe, B.; Bennett, R.; Keun, H.; Lindon, J.C.; Nicholson, J.K.; Dorff, M.H.; Jaroszewski, J.W.; et al. ^{1} H NMR Spectroscopy-Based Interventional Metabolic Phenotyping: A Cohort Study of Rheumatoid Arthritis Patients. *J. Proteome Res.* **2010**, *9*, 4545–4553.

6. Wu, Q.; Zhang, Q.; Sun, B.; Yan, X.; Tang, Y.; Qiao, X.; Chen, Q.; Yu, S.; Liang, F. 1H NMR-based metabonomic study on the metabolic changes in the plasma of patients with functional dyspepsia and the effect of acupuncture. *J. Pharm. Biomed. Anal.* **2010**, *51*, 698–704.

7. Hasim, A.; Ma, H.; Mamtimin, B.; Abudula, A.; Niyaz, M.; Zhang, L.; Anwer, J.; Sheyhidin, I. Revealing the metabonomic variation of EC using 1H-NMR spectroscopy and its association with the clinicopathological characteristics. *Mol. Biol. Rep.* **2012**, *39*, 8955–8964.

8. Hasim, A.; Ali, M.; Mamtimin, B.; Ma, J.; Li, G.; Abudula, A. Metabonomic signature analysis of cervical carcinoma and precancerous lesions in women by 1H NMR spectroscopy. *Exp. Ther. Med.* **2012**, *3*, 945–951.

9. Li, P.; Tao, J.; Wei, D.; Yang, X.; Lu, Z.; Deng, X.; Cheng, Y.; Gu, J.; Yang, X.; Wang, Z.; et al. Serum metabolomic analysis of human upper urinary

tract urothelial carcinoma. *Tumor Biol.* **2015**, *36*, 7531–7537.

10. Boguszewicz, Ł.; Hajduk, A.; Mrochem-Kwarciak, J.; Skorupa, A.; Ciszek, M.; Heyda, A.; Składowski, K.; Sokół, M. 1H NMR based metabolomic approach to monitoring of the head and neck cancer treatment toxicity. *Metabolomics* **2016**, *12*, 102.

11. Otvos, J.D.; Jeyarajah, E.J.; Bennett, D.W. Quantification of plasma lipoproteins by proton nuclear magnetic resonance spectroscopy. *Clin. Chem.* **1991**, *37*, 377–86.

12. Jeyarajah, E.J.; Cromwell, W.C.; Otvos, J.D. Lipoprotein Particle Analysis by Nuclear Magnetic Resonance Spectroscopy. *Clin. Lab. Med.* **2006**, *26*, 847–870.

13. Otvos, J.D.; Jeyarajah, E.J.; Bennett, D.W.; Krauss, R.M. Development of a proton nuclear magnetic resonance spectroscopic method for determining plasma lipoprotein concentrations and subspecies distributions from a single, rapid measurement. *Clin. Chem.* **1992**, *38*, 1632–8.

14. Otvos, J.D.; Shalaurova, I.; Wolak-Dinsmore, J.; Connelly, M.A.; Mackey, R.H.; Stein, J.H.; Tracy, R.P. GlycA: A Composite Nuclear Magnetic Resonance Biomarker of Systemic Inflammation. *Clin. Chem.* **2015**, *61*, 714–723.

15. Soininen, P.; Kangas, A.J.; Würtz, P.; Tukiainen, T.; Tynkkynen, T.; Laatikainen, R.; Järvelin, M.-R.; Kähönen, M.; Lehtimäki, T.; Viikari, J.; et al. High-throughput serum NMR metabonomics for cost-effective holistic studies on systemic metabolism †. *Analyst* **2009**, *134*, 1781–1785.

16. Ala-Korpela, M. 1H NMR spectroscopy of human blood plasma. *Prog. Nucl. Magn. Reson. Spectrosc.* **1995**, *27*, 475–554.

17. Soininen, P.; Kangas, A.J.; Würtz, P.; Suna, T.; Ala-Korpela, M. Quantitative Serum Nuclear Magnetic Resonance Metabolomics in Cardiovascular Epidemiology and Genetics. *Circ. Cardiovasc. Genet.* **2015**, *8*, 192–206.

18. Kujala, U.M.; Mäkinen, V.-P.; Heinonen, I.; Soininen, P.; Kangas, A.J.; Leskinen, T.H.; Rahkila, P.; Würtz, P.; Kovanen, V.; Cheng, S.; et al. Long-term Leisure-time Physical Activity and Serum Metabolome. *Circulation* **2013**, *127*, 340–348.

19. Bell, J.D.; Brown, J.C.C.; Nicholson, J.K.; Sadler, P.J. Assignment of resonances for 'acute-phase' glycoproteins in high resolution proton NMR spectra of human blood plasma. *FEBS Lett.* **1987**, *215*, 311–315.

20. De Meyer, T.; Sinnaeve, D.; Van Gasse, B.; Tsiporkova, E.; Rietzschel, E.R.;

De Buyzere, M.L.; Gillebert, T.C.; Bekaert, S.; Martins, J.C.; Van Criekinge, W. NMR-Based Characterization of Metabolic Alterations in Hypertension Using an Adaptive, Intelligent Binning Algorithm. *Anal. Chem.* **2008**, *80*, 3783–3790.

21. Correia, G.D.S.; Wooi Ng, K.; Wijeyesekera, A.; Gala-Peralta, S.; Williams, R.; MacCarthy-Morrogh, S.; Jiménez, B.; Inwald, D.; Macrae, D.; Frost, G.; et al. Metabolic Profiling of Children Undergoing Surgery for Congenital Heart Disease. *Crit. Care Med.* **2015**, *43*, 1467–1476.

22. Delaglio, F.; Grzesiek, S.; Vuister, G.; Zhu, G.; Pfeifer, J.; Bax, A. NMRPipe: A multidimensional spectral processing system based on UNIX pipes. *J. Biomol. NMR* **1995**, *6*, 277–293.

23. Lécuyer, L.; Victor Bala, A.; Deschasaux, M.; Bouchemal, N.; Nawfal Triba, M.; Vasson, M.-P.; Rossary, A.; Demidem, A.; Galan, P.; Hercberg, S.; et al. NMR metabolomic signatures reveal predictive plasma metabolites associated with long-term risk of developing breast cancer. *Int. J. Epidemiol.* **2018**, *47*, 484–494.

24. Jobard, E.; Pontoizeau, C.; Blaise, B.J.; Bachelot, T.; Elena-Herrmann, B.; Trédan, O. A serum nuclear magnetic resonance-based metabolomic signature of advanced metastatic human breast cancer. *Cancer Lett.* **2014**, *343*, 33–41.

25. Deja, S.; Porebska, I.; Kowal, A.; Zabek, A.; Barg, W.; Pawelczyk, K.; Stanimirova, I.; Daszykowski, M.; Korzeniewska, A.; Jankowska, R.; et al. Metabolomics provide new insights on lung cancer staging and discrimination from chronic obstructive pulmonary disease. *J. Pharm. Biomed. Anal.* **2014**, *100*, 369–380.

26. Huo, T.; Chen, X.; Lu, X.; Qu, L.; Liu, Y.; Cai, S. An effective assessment of valproate sodium-induced hepatotoxicity with UPLC–MS and 1HNMR-based metabonomics approach. *J. Chromatogr. B* **2014**, *969*, 109–116.

27. Fages, A.; Duarte-Salles, T.; Stepien, M.; Ferrari, P.; Fedirko, V.; Pontoizeau, C.; Trichopoulou, A.; Aleksandrova, K.; Tjønneland, A.; Olsen, A.; et al. Metabolomic profiles of hepatocellular carcinoma in a European prospective cohort. *BMC Med.* **2015**, *13*, 242.

28. Sands, C.J.; Guha, I.N.; Kyriakides, M.; Wright, M.; Beckonert, O.; Holmes, E.; Rosenberg, W.M.; Coen, M. Metabolic Phenotyping for Enhanced Mechanistic Stratification of Chronic Hepatitis C-Induced Liver Fibrosis. *Am. J. Gastroenterol.* **2015**, *110*, 159–169.

29. Tian, J.; Xia, X.; Wu, Y.; Zhao, L.; Xiang, H.; Du, G.; Zhang, X.; Qin, X. Discovery, screening and evaluation of a plasma biomarker panel for

subjects with psychological suboptimal health state using 1H-NMR-based metabolomics profiles. *Sci. Rep.* **2016**, *6*, 33820.

30. Jain, A.; Kumar, D.; Guleria, A.; Misra, D.P.; Zanwar, A.; Chaurasia, S.; Kumar, S.; Kumar, U.; Mishra, S.K.; Goel, R.; et al. NMR-Based Serum Metabolomics of Patients with Takayasu Arteritis: Relationship with Disease Activity. *J. Proteome Res.* **2018**, *17*, 3317–3324.

31. Suman, S.; Sharma, R.K.; Kumar, V.; Sinha, N.; Shukla, Y. Metabolic fingerprinting in breast cancer stages through 1H NMR spectroscopy-based metabolomic analysis of plasma. *J. Pharm. Biomed. Anal.* **2018**, *160*, 38–45.

32. Olivier Cloarec, †; Marc-Emmanuel Dumas, †; Andrew Craig, †; Richard H. Barton, †; Johan Trygg, ‡; Jane Hudson, §; Christine Blancher, §; Dominique Gauguier, §; John C. Lindon, †; Elaine Holmes, † and; et al. Statistical Total Correlation Spectroscopy: An Exploratory Approach for Latent Biomarker Identification from Metabolic 1H NMR Data Sets. *Anal. Chem* **2005**, *77*, 1282–1289.

33. Pinto, J.; Barros, A.S.; Domingues, M.R.M.; Goodfellow, B.J.; Galhano, E.; Pita, C.; Almeida, M. do C.; Carreira, I.M.; Gil, A.M. Following Healthy Pregnancy by NMR Metabolomics of Plasma and Correlation to Urine. *J. Proteome Res.* **2015**, *14*, 1263–1274.

34. Connelly, M.A.; Gruppen, E.G.; Wolak-Dinsmore, J.; Matyus, S.P.; Riphagen, I.J.; Shalaurova, I.; Bakker, S.J.L.; Otvos, J.D.; Dullaart, R.P.F. GlycA, a marker of acute phase glycoproteins, and the risk of incident type 2 diabetes mellitus: PREVEND study. *Clin. Chim. Acta* **2016**, *452*, 10–17.

35. Duprez, D.A.; Otvos, J.; Sanchez, O.A.; Mackey, R.H.; Tracy, R.; Jacobs, D.R. Comparison of the Predictive Value of GlycA and Other Biomarkers of Inflammation for Total Death, Incident Cardiovascular Events, Noncardiovascular and Noncancer Inflammatory-Related Events, and Total Cancer Events. *Clin. Chem.* **2016**, *62*, 1020–31.

36. Kelimu, A.; Xie, R.; Zhang, K.; Zhuang, Z.; Mamtimin, B.; Sheyhidin, I. Metabonomic signature analysis in plasma samples of glioma patients based on ^{1} H-nuclear magnetic resonance spectroscopy. *Neurol. India* **2016**, *64*, 246.

37. Chandler, P.D.; Akinkuolie, A.O.; Tobias, D.K.; Lawler, P.R.; Li, C.; Moorthy, M.V.; Wang, L.; Duprez, D.A.; Jacobs, D.R.; Glynn, R.J.; et al. Association of N-Linked Glycoprotein Acetyls and Colorectal Cancer Incidence and Mortality. *PLoS One* **2016**, *11*, e0165615.

38. Olson, M.L.; Rentería-Mexía, A.; Connelly, M.A.; Vega-López, S.; Soltero, E.G.; Konopken, Y.P.; Williams, A.N.; Castro, F.G.; Keller, C.S.; Yang, H.P.; et al. Decreased GlycA after lifestyle intervention among obese, prediabetic adolescent Latinos. *J. Clin. Lipidol.* **2018**.

39. Manmadhan, A.; Lin, B.-X.; Zhong, J.; Parikh, M.; Berger, J.S.; Fisher, E.A.; Heffron, S.P. Elevated GlycA in severe obesity is normalized by bariatric surgery. *Diabetes, Obes. Metab.* **2019**, *21*, 178–182.

40. Rawat, A.; Misra, G.; Saxena, M.; Tripathi, S.; Dubey, D.; Saxena, S.; Aggarwal, A.; Gupta, V.; Khan, M.Y.; Prakash, A. 1H NMR based serum metabolic profiling reveals differentiating biomarkers in patients with diabetes and diabetes-related complication. *Diabetes Metab. Syndr. Clin. Res. Rev.* **2019**, *13*, 290–298.

41. Akinkuolie, A.O.; Pradhan, A.D.; Buring, J.E.; Ridker, P.M.; Mora, S. Novel Protein Glycan Side-Chain Biomarker and Risk of Incident Type 2 Diabetes Mellitus. *Arterioscler. Thromb. Vasc. Biol.* **2015**, *35*, 1544–1550.

42. Houttu, N.; Mokkala, K.; Laitinen, K. Overweight and obesity status in pregnant women are related to intestinal microbiota and serum metabolic and inflammatory profiles. *Clin. Nutr.* **2018**, *37*, 1955–1966.

43. Fizelova, M.; Jauhiainen, R.; Kangas, A.J.; Soininen, P.; Ala-Korpela, M.; Kuusisto, J.; Laakso, M.; Stančáková, A. Differential Associations of Inflammatory Markers With Insulin Sensitivity and Secretion: The Prospective METSIM Study. *J. Clin. Endocrinol. Metab.* **2017**, *102*, 3600–3609.

44. Lorenzo, C.; Festa, A.; Hanley, A.J.; Rewers, M.J.; Escalante, A.; Haffner, S.M. Novel Protein Glycan–Derived Markers of Systemic Inflammation and C-Reactive Protein in Relation to Glycemia, Insulin Resistance, and Insulin Secretion. *Diabetes Care* **2017**, *40*, 375–382.

45. Bogl, L.H.; Kaye, S.M.; Rämö, J.T.; Kangas, A.J.; Soininen, P.; Hakkarainen, A.; Lundbom, J.; Lundbom, N.; Ortega-Alonso, A.; Rissanen, A.; et al. Abdominal obesity and circulating metabolites: A twin study approach. *Metabolism.* **2016**, *65*, 111–21.

46. Bartlett, D.B.; Slentz, C.A.; Connelly, M.A.; Piner, L.W.; Willis, L.H.; Bateman, L.A.; Granville, E.O.; Bales, C.W.; Huffman, K.M.; Kraus, W.E. Association of the Composite Inflammatory Biomarker GlycA, with Exercise-Induced Changes in Body Habitus in Men and Women with Prediabetes. *Oxid. Med. Cell. Longev.* **2017**, *2017*, 1–12.

47. Gruppen, E.G.; Connelly, M.A.; Vart, P.; Otvos, J.D.; Bakker, S.J.; Dullaart, R.P. GlycA, a novel proinflammatory glycoprotein biomarker, and high-

sensitivity C-reactive protein are inversely associated with sodium intake after controlling for adiposity: the Prevention of Renal and Vascular End-Stage Disease study. *Am. J. Clin. Nutr.* **2016**, *104*, 415–422.

48. Wurtz, P.; Tiainen, M.; Makinen, V.-P.; Kangas, A.J.; Soininen, P.; Saltevo, J.; Keinanen-Kiukaanniemi, S.; Mantyselka, P.; Lehtimaki, T.; Laakso, M.; et al. Circulating Metabolite Predictors of Glycemia in Middle-Aged Men and Women. *Diabetes Care* **2012**, *35*, 1749–1756.

49. Gruppen, E.G.; Connelly, M.A.; Otvos, J.D.; Bakker, S.J.L.; Dullaart, R.P.F. A novel protein glycan biomarker and LCAT activity in metabolic syndrome. *Eur. J. Clin. Invest.* **2015**, *45*, 850–859.

50. Dungan, K.; Binkley, P.; Osei, K. GlycA is a Novel Marker of Inflammation Among Non-Critically Ill Hospitalized Patients with Type 2 Diabetes. *Inflammation* **2015**, *38*, 1357–1363.

51. Dullaart, R.P.F.; Gruppen, E.G.; Connelly, M.A.; Lefrandt, J.D. A pro-inflammatory glycoprotein biomarker is associated with lower bilirubin in metabolic syndrome. *Clin. Biochem.* **2015**, *48*, 1045–1047.

52. Holmes, M. V.; Millwood, I.Y.; Kartsonaki, C.; Hill, M.R.; Bennett, D.A.; Boxall, R.; Guo, Y.; Xu, X.; Bian, Z.; Hu, R.; et al. Lipids, Lipoproteins, and Metabolites and Risk of Myocardial Infarction and Stroke. *J. Am. Coll. Cardiol.* **2018**, *71*, 620–632.

53. Muhlestein, J.B.; May, H.T.; Galenko, O.; Knowlton, K.U.; Otvos, J.D.; Connelly, M.A.; Lappe, D.L.; Anderson, J.L. GlycA and hsCRP are independent and additive predictors of future cardiovascular events among patients undergoing angiography: The intermountain heart collaborative study. *Am. Heart J.* **2018**, *202*, 27–32.

54. McGarrah, R.W.; Kelly, J.P.; Craig, D.M.; Haynes, C.; Jessee, R.C.; Huffman, K.M.; Kraus, W.E.; Shah, S.H. A Novel Protein Glycan–Derived Inflammation Biomarker Independently Predicts Cardiovascular Disease and Modifies the Association of HDL Subclasses with Mortality. *Clin. Chem.* **2017**, *63*, 288–296.

55. Muhlestein, J.B.; May, H.; Winegar, D.; Rollo, J.; Connelly, M.; Otvos, J.; Anderson, J. Differential association of high-density lipoprotein particle subclasses and GlycA, a novel inflammatory marker, in predicting cardiac death among patients undergoing angiography: the intermountain heart collaborative study. *J. Am. Coll. Cardiol.* **2016**, *67*, 162.

56. McGarrah, R.; Craig, D.; Haynes, C.; Dowdy, Z.E.; Shah, S.; Kraus, W. GlycA, a novel biomarker of systemic inflammation, improves cardiovascular risk prediction in a high-risk coronary catheterization

cohort. *J. Am. Coll. Cardiol.* **2015**, *65*, A1606.

57. Muhlestein, J.B.; May, H.; Winegar, D.; Rollo, J.; Connelly, M.; Otvos, J.; Anderson, J. GlycA and GlycB, novel NMR biomarkers of inflammation, strongly predict future cardiovascular events, but not the presence of coronary artery disease (CAD), among patients undergoing coronary angiography: the intermountain heart collaborative study. *J. Am. Coll. Cardiol.* **2014**, *63*, A1389.

58. Akinkuolie, A.O.; Buring, J.E.; Ridker, P.M.; Mora, S. A novel protein glycan biomarker and future cardiovascular disease events. *J. Am. Heart Assoc.* **2014**, *3*, e001221.

59. Fischer, K.; Kettunen, J.; Würtz, P.; Haller, T.; Havulinna, A.S.; Kangas, A.J.; Soininen, P.; Esko, T.; Tammesoo, M.-L.; Mägi, R.; et al. Biomarker Profiling by Nuclear Magnetic Resonance Spectroscopy for the Prediction of All-Cause Mortality: An Observational Study of 17,345 Persons. *PLoS Med.* **2014**, *11*, e1001606.

60. Gruppen, E.G.; Connelly, M.A.; Sluiter, W.J.; Bakker, S.J.L.; Dullaart, R.P.F. Higher plasma GlycA, a novel pro-inflammatory glycoprotein biomarker, is associated with reduced life expectancy: The PREVEND study. *Clin. Chim. Acta* **2019**, *488*, 7–12.

61. Otvos, J.D.; Guyton, J.R.; Connelly, M.A.; Akapame, S.; Bittner, V.; Kopecky, S.L.; Lacy, M.; Marcovina, S.M.; Muhlestein, J.B.; Boden, W.E. Relations of GlycA and lipoprotein particle subspecies with cardiovascular events and mortality: A post hoc analysis of the AIM-HIGH trial. *J. Clin. Lipidol.* **2018**.

62. Benson, E.-M.A.; Tibuakuu, M.; Zhao, D.; Akinkuolie, A.O.; Otvos, J.D.; Duprez, D.A.; Jacobs, D.R.; Mora, S.; Michos, E.D. Associations of ideal cardiovascular health with GlycA, a novel inflammatory marker: The Multi-Ethnic Study of Atherosclerosis. *Clin. Cardiol.* **2018**, *41*, 1439–1445.

63. Ritchie, S.C.; Kettunen, J.; Brozynska, M.; Nath, A.P.; Havulinna, A.S.; Männistö, S.; Perola, M.; Salomaa, V.; Ala-Korpela, M.; Abraham, G.; et al. Elevated alpha-1 antitrypsin is a major component of GlycA-associated risk for future morbidity and mortality. *bioRxiv* **2018**, 309138.

64. Lawler, P.R.; Akinkuolie, A.O.; Chandler, P.D.; Moorthy, M.V.; Vandenburgh, M.J.; Schaumberg, D.A.; Lee, I.-M.; Glynn, R.J.; Ridker, P.M.; Buring, J.E.; et al. Circulating N-Linked Glycoprotein Acetyls and Longitudinal Mortality Risk. *Circ. Res.* **2016**, *118*, 1106–1115.

65. Tibuakuu, M.; Fashanu, O.E.; Bs, M.B.; Zhao, D.; Otvos, J.D.; Brown, T.T.; Haberlen, S.A.; Guallar, E.; Budoff, M.J.; Palella, F.J.; et al. GlycA, a Novel

Inflammatory Marker, is Associated with Subclinical Coronary Disease in the Multicenter AIDS Cohort Study Short title: GlycA and Coronary Plaque in HIV. *UCLA Previously Publ. Work.* **2018**.

66. Dierckx, T.; Verstockt, B.; Vermeire, S.; van Weyenbergh, J. GlycA, a Nuclear Magnetic Resonance Spectroscopy Measure for Protein Glycosylation, is a Viable Biomarker for Disease Activity in IBD. *J. Crohn's Colitis* **2018**, 1–6.

67. Fuertes-Martín, R.; Taverner, D.; Vallvé, J.-C.; Paredes, S.; Masana, L.; Correig Blanchar, X.; Amigó Grau, N. Characterization of ^{1} H NMR Plasma Glycoproteins as a New Strategy To Identify Inflammatory Patterns in Rheumatoid Arthritis. *J. Proteome Res.* **2018**, *17*, 3730–3739.

68. Dierckx, T.; Goletti, S.; Chiche, L.; Daniel, L.; Lauwerys, B.; Jourde-Chiche, N.; Weyenbergh, J. Van Serum GlycA level is a candidate biomarker for disease activity in systemic lupus erythematosus and for proliferative status of lupus nephritis, independent of renal function impairment. *bioRxiv* **2018**, 493809.

69. Xu, W.; Upur, H.; Wu, Y.; Mamtimin, B.; Yang, J.; Ga, Y.; You, L. Metabolomic changes in patients with chronic obstructive pulmonary disease with abnormal Savda syndrome. *Exp. Ther. Med.* **2015**, *9*, 425–431.

70. Titan, S.M.; Pecoits-Filho, R.; Barreto, S.M.; Lopes, A.A.; Bensenor, I.J.; Lotufo, P.A. GlycA, a marker of protein glycosylation, is related to albuminuria and estimated glomerular filtration rate: the ELSA-Brasil study. *BMC Nephrol.* **2017**, *18*, 367.

71. Durcan, L.; Winegar, D.A.; Connelly, M.A.; Otvos, J.D.; Magder, L.S.; Petri, M. Longitudinal Evaluation of Lipoprotein Variables in Systemic Lupus Erythematosus Reveals Adverse Changes with Disease Activity and Prednisone and More Favorable Profiles with Hydroxychloroquine Therapy. *J. Rheumatol.* **2016**, *43*, 745–750.

72. Joshi, A.A.; Lerman, J.B.; Aberra, T.M.; Afshar, M.; Teague, H.L.; Rodante, J.A.; Krishnamoorthy, P.; Ng, Q.; Aridi, T.Z.; Salahuddin, T.; et al. GlycA Is a Novel Biomarker of Inflammation and Subclinical Cardiovascular Disease in Psoriasis. *Circ. Res.* **2016**, *119*.

73. Bartlett, D.B.; Connelly, M.A.; AbouAssi, H.; Bateman, L.A.; Tune, K.N.; Huebner, J.L.; Kraus, V.B.; Winegar, D.A.; Otvos, J.D.; Kraus, W.E.; et al. A novel inflammatory biomarker, GlycA, associates with disease activity in rheumatoid arthritis and cardio-metabolic risk in BMI-matched controls. *Arthritis Res. Ther.* **2016**, *18*, 86.

74. Ormseth, M.J.; Chung, C.P.; Oeser, A.M.; Connelly, M.A.; Sokka, T.;

Raggi, P.; Solus, J.F.; Otvos, J.D.; Stein, C.M. Utility of a novel inflammatory marker, GlycA, for assessment of rheumatoid arthritis disease activity and coronary atherosclerosis. *Arthritis Res. Ther.* **2015**, *17*, 117.

75. Chung, C.P.; Ormseth, M.J.; Connelly, M.A.; Oeser, A.; Solus, J.F.; Otvos, J.D.; Raggi, P.; Stein, C.M. GlycA, a novel marker of inflammation, is elevated in systemic lupus erythematosus. *Lupus* **2016**, *25*, 296–300.

76. Ritchie, S.C.; Würtz, P.; Nath, A.P.; Abraham, G.; Havulinna, A.S.; Fearnley, L.G.; Sarin, A.-P.; Kangas, A.J.; Soininen, P.; Aalto, K.; et al. The Biomarker GlycA Is Associated with Chronic Inflammation and Predicts Long-Term Risk of Severe Infection. *Cell Syst.* **2015**, *1*, 293–301.

77. Tukiainen, T.; Tynkkynen, T.; Mäkinen, V.-P.; Jylänki, P.; Kangas, A.; Hokkanen, J.; Vehtari, A.; Gröhn, O.; Hallikainen, M.; Soininen, H.; et al. A multi-metabolite analysis of serum by 1H NMR spectroscopy: Early systemic signs of Alzheimer's disease. *Biochem. Biophys. Res. Commun.* **2008**, *375*, 356–361.

78. Cohen-Manheim, I.; Doniger, G.M.; Sinnreich, R.; Simon, E.S.; Pinchas-Mizrachi, R.; Otvos, J.D.; Kark, J.D. Increase in the Inflammatory Marker GlycA over 13 Years in Young Adults Is Associated with Poorer Cognitive Function in Midlife. *PLoS One* **2015**, *10*, e0138036.

79. Connelly, M.A.; Shimizu, C.; Winegar, D.A.; Shalaurova, I.; Pourfarzib, R.; Otvos, J.D.; Kanegaye, J.T.; Tremoulet, A.H.; Burns, J.C. Differences in GlycA and lipoprotein particle parameters may help distinguish acute kawasaki disease from other febrile illnesses in children. *BMC Pediatr.* **2016**, *16*.

80. Guleria, A.; Misra, D.P.; Rawat, A.; Dubey, D.; Khetrapal, C.L.; Bacon, P.; Misra, R.; Kumar, D. NMR-Based Serum Metabolomics Discriminates Takayasu Arteritis from Healthy Individuals: A Proof-of-Principle Study. *J. Proteome Res.* **2015**, *14*, 3372–3381.

81. Röytiö, H.; Mokkala, K.; Vahlberg, T.; Laitinen, K. Dietary intake of fat and fibre according to reference values relates to higher gut microbiota richness in overweight pregnant women. *Br. J. Nutr.* **2017**, *118*, 343–352.

82. Mokkala, K.; Pellonperä, O.; Röytiö, H.; Pussinen, P.; Rönnemaa, T.; Laitinen, K. Increased intestinal permeability, measured by serum zonulin, is associated with metabolic risk markers in overweight pregnant women. *Metabolism* **2017**, *69*, 43–50.

83. Berends, A.M.A.; Buitenwerf, E.; Gruppen, E.G.; Sluiter, W.J.; Bakker, S.J.L.; Connelly, M.A.; Kerstens, M.N.; Dullaart, R.P.F. Primary

aldosteronism is associated with decreased low-density and high-density lipoprotein particle concentrations and increased GlycA, a pro-inflammatory glycoprotein biomarker. *Clin. Endocrinol. (Oxf).* **2019**, *90*, 79–87.

84. Weisman, J.K.; Meeks, D.; Mendelsohn, L.; Remaley, A.T.; Sampson, M.; Allen, D.T.; Nichols, J.; Shet, A.S.; Thein, S.L. GlycA is not a useful biomarker of inflammation in sickle cell disease. *Int. J. Lab. Hematol.* **2018**, *40*, 704–709.

85. Lamour, S.D.; Gomez-Romero, M.; Vorkas, P.A.; Alibu, V.P.; Saric, J.; Holmes, E.; Sternberg, J.M. Discovery of Infection Associated Metabolic Markers in Human African Trypanosomiasis. *PLoS Negl. Trop. Dis.* **2015**, *9*, e0004200.

86. Gruppen, E.G.; Connelly, M.A.; Dullaart, R.P.F. Higher circulating GlycA, a pro-inflammatory glycoprotein biomarker, relates to lipoprotein-associated phospholipase A2 mass in nondiabetic subjects but not in diabetic or metabolic syndrome subjects. *J. Clin. Lipidol.* **2016**, *10*, 512–518.

87. Kianoush, S.; Bittencourt, M.S.; Lotufo, P.A.; Bensenor, I.M.; Jones, S.R.; DeFilippis, A.P.; Toth, P.P.; Otvos, J.D.; Tibuakuu, M.; Hall, M.E.; et al. Association Between Smoking and Serum GlycA and High-Sensitivity C-Reactive Protein Levels: The Multi-Ethnic Study of Atherosclerosis (MESA) and Brazilian Longitudinal Study of Adult Health (ELSA-Brasil). *J. Am. Heart Assoc.* **2017**, *6*.

88. Barber, J.L.; Kraus, W.E.; Church, T.S.; Hagberg, J.M.; Thompson, P.D.; Bartlett, D.B.; Beets, M.W.; Earnest, C.P.; Huffman, K.M.; Landers-Ramos, R.Q.; et al. Effects of regular endurance exercise on GlycA: Combined analysis of 14 exercise interventions. *Atherosclerosis* **2018**, *277*, 1–6.

89. Kelesidis, T.; Tran, T.T.T.; Stein, J.H.; Brown, T.T.; Moser, C.; Ribaudo, H.J.; Dube, M.P.; Murphy, R.; Yang, O.O.; Currier, J.S.; et al. Changes in Inflammation and Immune Activation With Atazanavir-, Raltegravir-, Darunavir-Based Initial Antiviral Therapy: ACTG 5260s. *Clin. Infect. Dis.* **2015**, *61*, 651–660.

90. Dullaart, R.P.F.; Gruppen, E.G.; Connelly, M.A.; Otvos, J.D.; Lefrandt, J.D. GlycA, a biomarker of inflammatory glycoproteins, is more closely related to the leptin/adiponectin ratio than to glucose tolerance status. *Clin. Biochem.* **2015**, *48*, 811–814.

91. Jago, R.; Drews, K.L.; Otvos, J.D.; Willi, S.M.; Buse, J.B. Novel measures of inflammation and insulin resistance are related to obesity and fitness in a diverse sample of 11–14 year olds: The HEALTHY Study. *Int. J. Obes.* **2016**, *40*, 1157–1163.
